# 2D Inorganic Electrides: Interstitial Electrons as Key Drivers of Multifunctional Properties and Applications

**DOI:** 10.1002/advs.76098

**Published:** 2026-06-12

**Authors:** Qianwen Zhang, Xia Cheng, Zhenzhou Guo, Weiyao Jia, Ying Yang, Zhenxiang Cheng, Weizhen Meng

**Affiliations:** ^1^ School of Physical Science and Technology Southwest University Chongqing China; ^2^ Institute For Superconducting and Electronic Materials Faculty of Engineering and Information Sciences University of Wollongong Wollongong Australia; ^3^ College of Physics and Electronic Engineering Chongqing Normal University Chongqing China; ^4^ College of Physics Hebei Key Laboratory of Photophysics Research and Application Hebei Normal University Shijiazhuang China

**Keywords:** electronegativity, energy conversion efficiency, energy transformation, layered structure, materials science, monolayer, nanotechnology, spintronics, topological insulator, transition metal

## Abstract

2D inorganic electrides have attracted extensive interdisciplinary interest due to their unique physicochemical properties, which arise from the presence of non‐nuclear‐bound interstitial anionic electrons (IAEs). Nevertheless, the stringent design criteria for realizing 2D IAEs have limited viable candidates to only a few categories, thereby constraining the expansion of the candidate pool and further exploration of related applications. Here, based on the electronegativity differences of elements and combined with the design principles of 2D inorganic electrides, we constructed eight negative‐valence transition metal‐based AB‐type 2D inorganic electrides (A = Ca/Sr/Ba, B = Cu/Ag/Au). These materials exhibit a typical layered structure, forming an ordered alternating arrangement of atomic layers and IAE layers along the k_z_ direction. Interestingly, despite the absence of conventional magnetic atoms, their monolayer structures display distinct magnetic ordering—originating from surface‐floating IAEs. Furthermore, these electrides exhibit diverse topological phases and ultralow work functions. Leveraging their ability to mitigate hydrogen poisoning, we demonstrate that Ru supported on these electrides can serve as an efficient catalyst for ammonia (NH_3_) synthesis. These findings not only establish a new material platform for exploring 2D inorganic electrides but also open avenues for designing and modulating their multifunctional properties toward applications in spintronics, topological electronics, and energy conversion.

## Graphical abstract

Eight AB‐type 2D inorganic electrides with negatively charged transition metals are theoretically designed. Their monolayers exhibit intrinsic magnetic ordering, rich topological phases, and ultralow work functions. Supported metal Ru on these 2D inorganic electrides effectively alleviates hydrogen poisoning and enables efficient ammonia synthesis, expanding the 2D inorganic electrides family and offering promising platforms for spintronics and energy conversion.

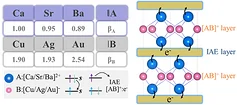

## 1 Introduction

Inorganic electrides have attracted considerable interest due to their distinctive structural geometries and electronic behaviors, which arise from highly localized interstitial anion electrons (IAEs) acting as quasi‐atoms within the cation‐formed cavities [1, 2, 3, 4]. These non‐nuclear‐bound IAEs endow inorganic electrides with a range of intriguing properties, including low work functions (WF), topological quantum states, and high‐pressure superconductivity [5, 6, 7, 8, 9, 10, 11]. Interestingly, Qi et al. [12] discovered an anomalous tribological property in the 2D inorganic electride Be_2_N, characterized by the coexistence of high interlayer adhesion energy and ultralow sliding energy barrier. Recent advances have demonstrated that single‐ or few‐layer structures offer remarkable advantages in tuning functional properties, driving a paradigm shift in inorganic electrides from bulk materials toward 2D van der Waals (VDW) systems [13, 14]. Remarkably, Such VDW inorganic electrides not only preserve key physical properties of their bulk counterparts but also give rise to emergent phenomena—such as floating surface electron states [15, 16, 17] and Stoner‐instability‐induced surface magnetism [18, 19, 20]. These distinctive properties indicate promising applications in spintronic devices and energy storage and conversion technologies—including ammonia (NH_3_) synthesis and the hydrogen evolution reaction [21–23].

Parallel to these advances, topological semimetals have gained prominence due to their robust topological surface states, which contribute to high carrier mobility and enhanced surface reactivity, thereby positioning them at the forefront of condensed matter physics [24, 25, 26]. Recently, topological quantum chemistry and related theories have demonstrated that the topological properties near the Fermi level originate from weakly bound orbital electrons [27, 28]. This mechanism aligns remarkably well with the defining characteristics of metallic inorganic electrides, where non‐nuclear‐bound IAEs dominate the low‐energy physical properties [5]. Consequently, inorganic electrides have emerged as a highly promising platform for exploring topological states—a prospect that has already been demonstrated in various bulk and 2D systems, such as [Sr_3_CrN_3_]^+^:e^−^, [CeH_2_]^+^:e^−^, and [Sc_2_C]^2+^:2e^−^ [5, 29, 30, 31, 32]. Furthermore, the introduction of magnetism significantly enhances the tunability of topological states in these systems [29, 31, 32, 33]. A prominent example is the 2D inorganic electride [Gd_2_C]^2+^:2e^−^ [16, 32], where ferromagnetic (FM) order induces a relatively large and tunable anomalous Hall conductivity. These advances underscore the importance of constructing and expanding the family of 2D magnetic topological inorganic electrides.

Despite considerable progress in predicting 2D inorganic electrides via high‐throughput screening and inverse design strategies [33, 34, 35, 36], current research on 2D inorganic electrides has primarily focused on main group II nitrides and rare‐earth‐based compounds such as carbides, oxides, and chlorides. Similarly, most reported magnetic inorganic electrides also originate from rare‐earth‐based systems. In contrast, 2D magnetic inorganic electrides based on transition metals have yet to be systematically explored.

In this work, guided by established design principles for 2D inorganic electrides—and leveraging elemental electronegativity differences—we designed eight AB‐type 2D inorganic electrides featuring negatively charged transition metals (TMs) (A = Ca, Sr, Ba; B = Cu, Ag, Au). Subsequently, we focused on the monolayer structures of these systems to systematically investigate their magnetism, topological states, WFs, and potential applications in NH_3_ synthesis. Our results reveal that the monolayer inorganic electrides exhibit diverse magnetic configurations, including five that exhibit FM ordering and two antiferromagnetic (AFM) structures. Furthermore, by passivating the surface IAEs with H atoms, a transition from magnetic to non‐magnetic (NM) behavior is achieved, confirming that the magnetism originates from the IAEs. Moreover, these monolayer inorganic electrides host various types of topological states, with corresponding Fermi‐arc edge states clearly observed. Notably, these monolayer structures also display ultralow WFs and, after loading with Ru, demonstrate effective applicability in NH_3_ synthesis. These findings provide an ideal platform for exploring 2D inorganic electrides and their related properties and applications.

## 2 Results and Discussion

### 2.1 Design and Material Realization of Negatively Charged TMs‐Based 2D Inorganic Electrides

**FIGURE 1:**
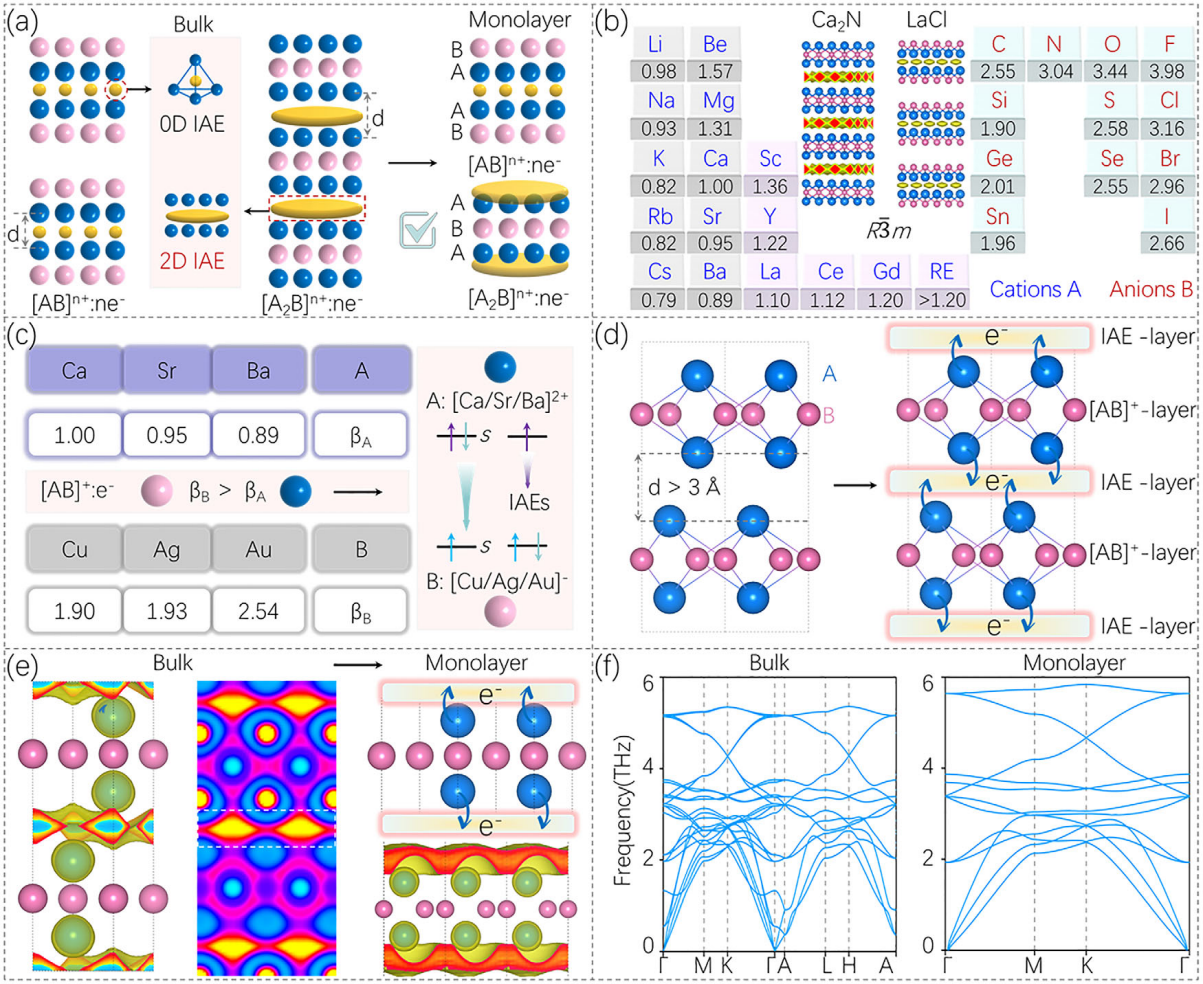
(a) Structural models of bulk and monolayer for AB‐ and A_2_B‐type van der Waals inorganic electrides. (b) The currently known binary 2D inorganic electrides consist of electropositive (blue) cations and electronegative (red) anions; the insets are ELF of two representative materials, Ca_2_N and LaCl. (c) The electronegativities (β_A_ and β_B_) of the transition metals B and the main‐group II elements A, along with the resulting charge transfer analysis driven by their electronegativity difference. (d) AB‐type van der Waals inorganic electrides composed of elements in (c). (e) ELF isosurfaces at a value of 0.55 for both bulk and monolayer structures within the AB‐type inorganic electrides. (f) Phonon spectra of bulk and monolayer AB inorganic electride family.

According to the established principles of 2D inorganic electrides [34], three fundamental criteria are generally required (see Figure 1a): (i) the system possesses intrinsic electron‐rich behavior, manifested as positively charged atomic layers; (ii) the structure exhibits a pronounced layered feature; and (iii) cations should be distributed on both sides of the interlayer region. Only when all these conditions are simultaneously satisfied can the IAEs be stabilized within the interlayer region, forming an ordered alternating arrangement of atomic layers and IAE layers along specific crystallographic directions. Notably, the interlayer spacing (*d*) plays a critical role in governing the dimensionality of the IAEs. When the *d* is approximately 3 Å, the IAEs tend to adopt a 2D distribution—exemplified by the [Ca_2_N]^+^:e^−^ family [34]. Conversely, if the *d* is too small, the IAEs may exhibit 0D characteristics—as observed in the [LaCl]^2+^:2e^−^ [14]—or even vanish entirely. Moreover, layered inorganic electrides with a relatively large *d* can be more readily exfoliated into monolayer systems. In such cases, the IAEs will float on the surface, exhibiting quasi‐2D electron gas (2DEG) behavior [35].

Currently identified 2D inorganic electrides are primarily classified within the *R* $\tilde{3}$ *m* space group (SG), with most existing as binary compounds of the AB and A_2_B types, as summarized in Table SI. As shown in Figure 1b, in terms of elemental composition, the cations (A) are generally derived from main group I/II elements, as well as light and heavy rare‐earth elements. Conversely, the anions (B) are mainly composed of elements from main groups IV→VII. Although some preliminary studies have explored TMs‐based 2D inorganic electrides (e.g., [Hf_2_S]^2+^:2e^−^ [37]), the TMs in these systems typically adopt positive valence states. Remarkably, TMs generally exhibit lower electronegativity than main group I/II elements. By leveraging this property and integrating it with the design principles of 2D inorganic electrides, rational tuning of the ratio between TMs and main group I/II elements in compositional design can enable TMs to exist in a negative valence state within 2D inorganic electrides.

As shown in Figure 1c, main group‐II Ca/Sr/Ba (denoted as A) and TMs Cu/Ag/Au (denoted as B) were selected as promising candidate elements, guided by the interstitial quasi‐atom model [38]. First, the electronegativity of TMs B is significantly higher than that of main group‐II elements A, which promotes the transfer of *s^2^*‐orbital electrons from A to B, thereby destabilizing the *s^1^*‐orbital electrons of A. Second, if TMs B and main group‐II elements A can form an AB‐type structure that satisfies the design principles of 2D inorganic electrides, the remaining single electron in the *s^1^*‐orbital electrons from A will be transferred to the interlayer region, forming a 2D IAEs. This design will directly enable the realization of negatively charged TMs‐based 2D inorganic electrides. Subsequently, we constructed eight 2[AB]^+^:2e^−^ (A = Ca, Sr, Ba; B = Cu, Ag, Au) models with a [Ca_2_N]^+^:e^−^‐like structure (belong to SG: *P6_3_/mmc*), excluding the already discovered inorganic electride BaCu [39] (see Figure 1d; Figure S1). Further electron localization function (ELF) analysis confirms the presence of highly localized 2D IAEs in the interlayer region of bulk inorganic electrides AB (see Figure 1e; Figure S2). Additionally, these candidate materials possess relatively large interlayer spacings *d*, and the 2D IAEs are found to persist even in exfoliated monolayer systems (see Figure 1e; Figure S3). However, phonon spectrum calculations reveal that among the eight candidate systems (an example of stable BaAg is shown in Figure 1f), CaAg and CaAu are unstable in both bulk and monolayer structures (see Figures S4 and S5). To further evaluate the thermodynamic stability of six stable 2D systems, we performed Ab initio Molecular Dynamics (AIMD) at 300 K (see Figure S6). Based on the entire energy evolution and final‐state structures, the lattices exhibit only slight deformation, indicating thermodynamic stability.

**FIGURE 2:**
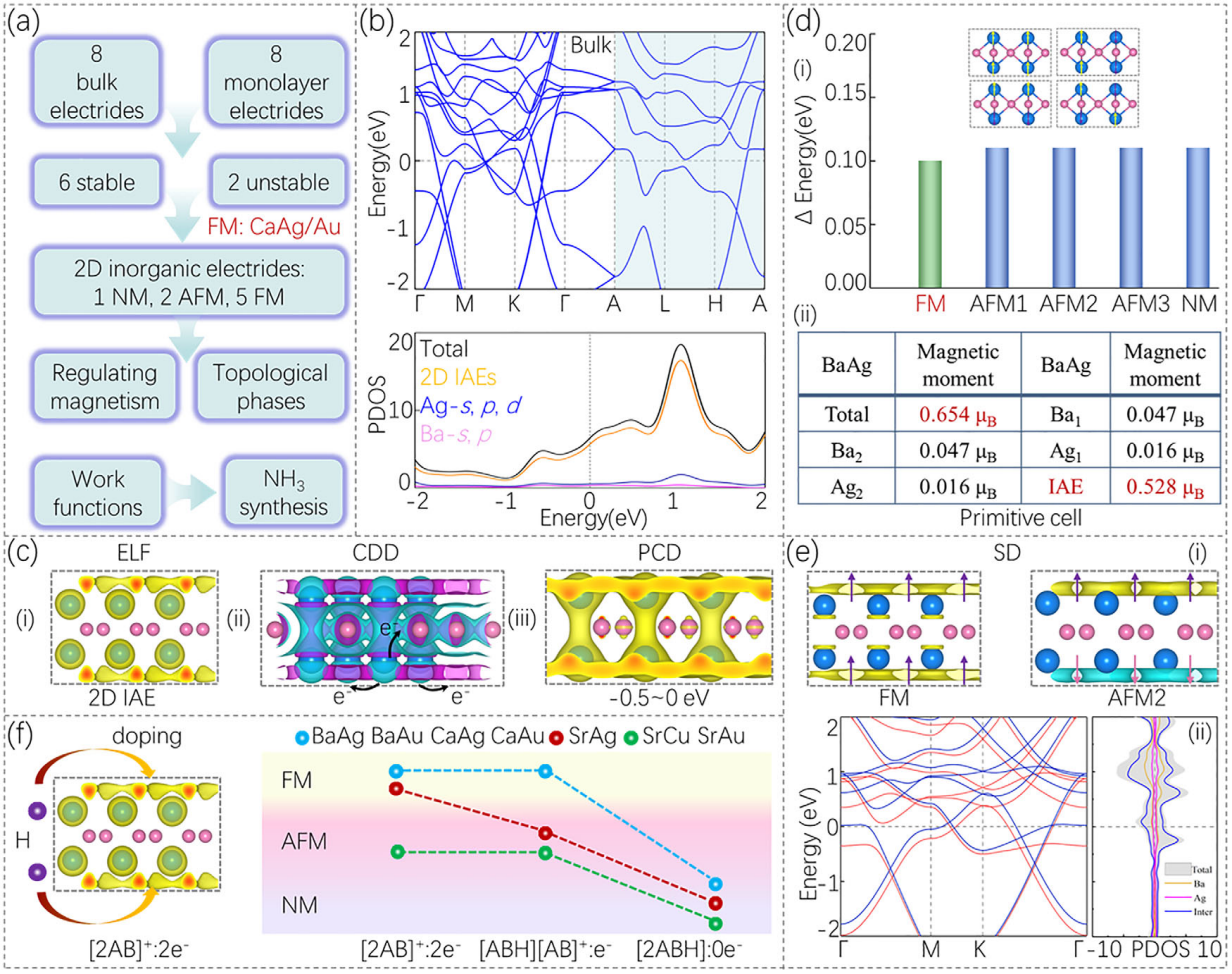
(a) Flowchart of the multifunctional properties and applications of eight AB‐type inorganic electrides. (b) The electronic band structure and PDOS of the bulk inorganic electride BaAg. (c) (i) The electron localization function (ELF), (ii) charge difference density, and (iii) partial charge density of the monolayer BaAg. (d) (i) The magnetic ground state of the monolayer BaAg and (ii) the corresponding magnetic moments of the atoms and IAEs. (e) (i) The spin density (SD) of monolayer BaAg in FM and AFM2 configurations, (ii) as well as the electronic band structure and PDOS in the FM ground state. (f) Magnetic phase transitions driven by neutralization of top‐layer and bottom‐layer IAEs with an H atom in monolayer AB‐family.

Unlike their bulk counterparts, all eight monolayer inorganic electrides—including two metastable candidates, CaAu and CaAg—exhibit relatively large magnetic moments under FM ordering (see Table SII). Notably, although CaCu possesses a magnetic moment of 0.34 *µ_B_* under FM ordering, its NM state energy is the lowest. Among the remaining five stable magnetic monolayer electrides (BaAg, BaAu, SrCu, SrAg, and SrAu), three display FM ordering (BaAg, BaAu, and SrAg), whereas the other two exhibit AFM behavior (SrCu and SrAu) (see Figure S7). Subsequently, we investigated their magnetic properties, topological phases, WF, and catalytic activity for NH_3_ synthesis in these monolayer systems, as shown in Figure 2a. In addition, specific computational details, approximate methods for calculating the magnetic ground state, and potential synthetic possibilities are presented in Section S1.

### 2.2 Magnetic Properties in 2D Inorganic Electrides

First, we found that eight bulk inorganic electrides exhibit NM behavior. Moreover, all systems demonstrate metallic characteristics (see Figure S8). Taking bulk inorganic electride BaAg as a representative example (see Figure 2b), further analysis of the partial density of states (PDOS) reveals that the electronic states near the Fermi level (−2 to 2 eV) are almost entirely contributed by the 2D IAEs, which is in excellent agreement with the results obtained from ELF analysis. Interestingly, the monolayer systems derived from bulk structures composed of NM atoms exhibit distinct magnetic configurations. Subsequently, using monolayer inorganic electride BaAg as an example, we discussed the magnetic‐related properties, covering the magnetic ground state, origin of magnetism, magnetoelectronic structure, and magnetic phase transitions.

The ELF of monolayer BaAg reveals the presence of IAEs localized above the surface, resembling a 2DEG, as shown in Figure 2c‐i. Charge density difference analysis further indicates that, besides charge transfer from Ba cations to Ag anions, substantial additional charge is transferred toward the surface (see Figure 2c‐ii). This finding supports the design rationale for 2D inorganic electrides based on electronegativity differences. Magnetic ground‐state energy comparisons indicate that the FM order is more stable, as shown in Figure 2d‐i. Notably, the total magnetic moment of monolayer BaAg in the FM state reaches 0.654 *µ_B_*, with only approximately 0.126 *µ_B_* distributed in all atoms—comprising two Ba and two Ag atoms in the primitive cell—while the surface 2D IAEs contribute a significant magnetic moment of 0.528 *µ_B_*. (see Figure 2d‐ii). Furthermore, the spin density (SD) distribution clearly shows that spin electrons are highly concentrated at the surface, which is fully consistent with the aforementioned magnetic moment analysis (see Figure 2e‐i). The electronic band structure indicates that 2D inorganic electride BaAg exhibits metallic behavior in the FM ground state; both the PDOSs and the partial charge density within the energy range of −0.5 to 0 eV reveal that the bands near the Fermi level are dominated by the surface 2D IAEs (see Figure 2c‐iii,e‐ii).

Due to the high reactivity of the exposed magnetic IAEs on the surface, it readily adsorbs various molecules and atoms. Currently, hydrogenation is one of the most effective methods for regulating the properties of IAEs in monolayer inorganic electrides, with rare‐earth‐based carbides (e.g., Gd_2_C [19]) serving as typical experimental examples [18, 19, 20]. For monolayer inorganic electride BaAg, the two IAEs are distributed on the top and bottom surfaces, respectively, as shown in Figure 2f. When only the top‐layer IAEs are hydrogenated, the system maintains FM order due to the retention of the bottom‐layer IAEs, but its magnetic moment is significantly reduced (see Table SIII). Upon further hydrogenation of the bottom‐layer IAEs, the magnetism of the system completely disappears. Additionally, both BaAgH and BaAgH_2_ retain metallic electronic structures. These results further confirm that the magnetism in monolayer BaAg primarily originates from IAEs. Similar trends are observed in other monolayer systems (see Figures S9 and S10 and Table SIII); however, in monolayer SrAg, hydrogenation of only the top‐layer IAEs induces a transition from FM to AFM states (see Figure 2f).

### 2.3 Topological States and WFs in 2D Inorganic Electrides

**FIGURE 3:**
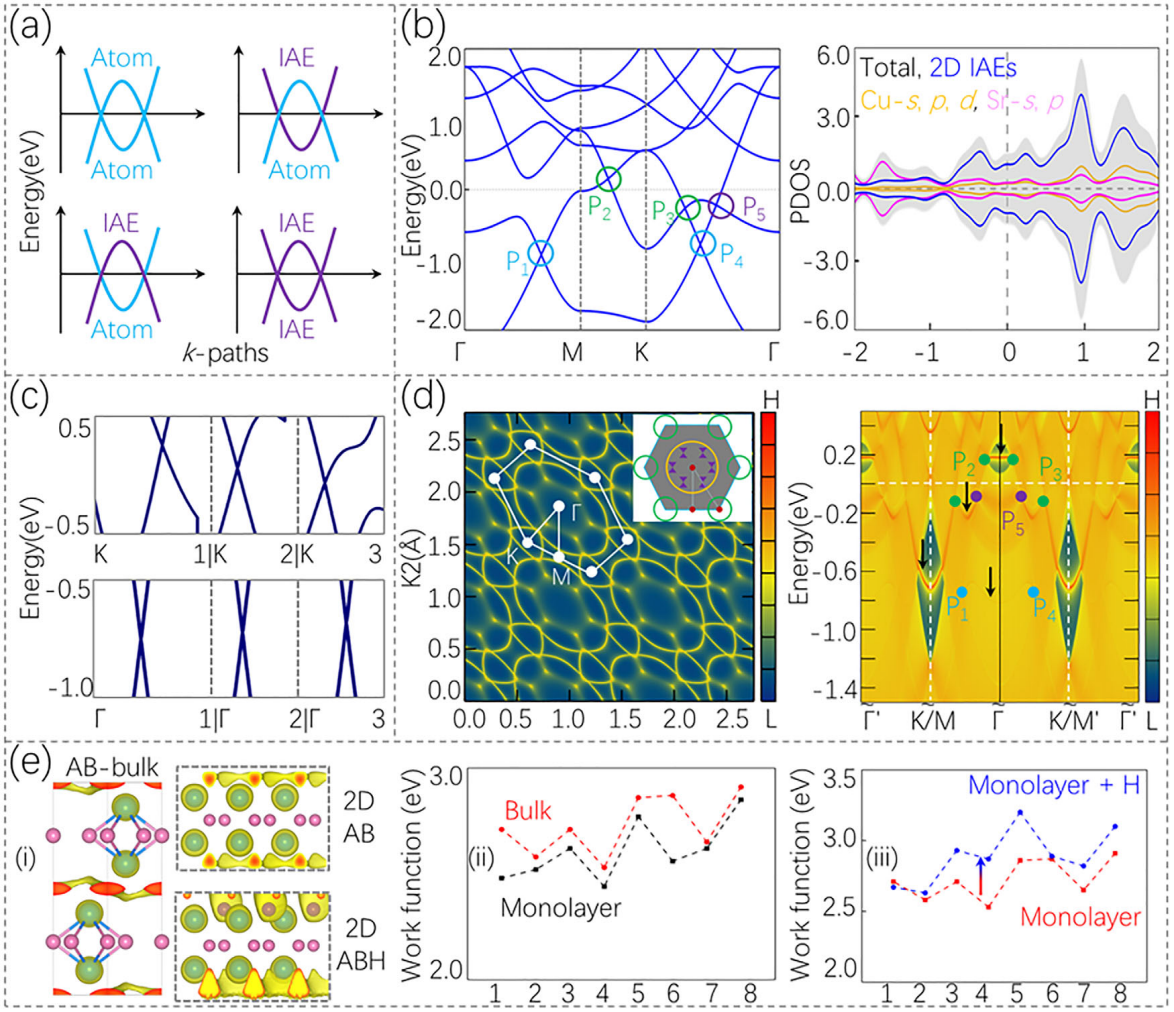
(a) Classification of crossing points at the Fermi level in inorganic electrides. (b) The electronic band structure and PDOS of the monolayer inorganic electride SrCu. (c) Verify the electronic band structures around the node loops of Γ and K points. Among them, 1, 2, and 3 are three k‐points on the K‐M and Γ‐K paths, respectively. (d) The left panel: the Fermi surface at E = −0.1 eV; the inset shows the positions of the topological states in the Brillouin zone. The right panel: the boundary state along the (010) direction. (e) ELF of bulk AB, monolayer AB, and ABH. (ii) and (iii) show the WFs along the (001) direction corresponding to (i), respectively. Among them, the serial number is Figure S1.

Inorganic electrides are widely regarded as ideal platforms for topological states. In such materials, the band crossings near the Fermi level generally exhibit three basic forms: those arising purely from atomic orbital electrons, those resulting from hybridization between atomic orbital electrons and IAEs, and those dominated entirely by IAEs, as shown in Figure 3a. In practice, PDOS analyses of real inorganic electrides reveal that the electronic structure near the Fermi level is rarely purely derived from IAEs; rather, contributions from both orbital electrons and IAEs are present, with the latter constituting a dominant role. Moreover, although the band structures of the eight bulk inorganic electrides are not optimal in topological investigations, the combined symmetry operation [*TS_z_*] —where *T* denotes time‐reversal symmetry and *S_z_* denotes screw rotation symmetry—enforces doubly degenerate bands across the entire k_z_ = 0 plane. This indicates that these bulk systems can be classified as surface semimetals [40].

Next, we turn our attention to monolayer systems. Given their shared band‐structure characteristics, we select the AFM monolayer SrCu as a representative case to explore its topological properties. As shown in Figure 3b, within the energy window above −1 eV, the electronic bands are primarily contributed by IAEs. Several band crossing points (CPs) are observed near the Fermi level and are labeled P_1_‐P_5_. According to the computational verification in Figure 3c, CPs P_2_ and P_3_ form a nodal ring (NR) around the K point, whereas CPs P_1_ and P_4_ constitute another NR around the Γ point; CP P_5_ emerges as an isolated Weyl point. Notably, the Fermi surfaces associated with the Γ‐ and K‐centered NRs exhibit hexagonal and triangular warping, respectively—signifying pronounced momentum‐space anisotropy in the system [41, 42] (see Figure 3d). The right panel of Figure 3d presents the boundary state projected onto the (010) direction. Distinct Fermi‐arc states—topologically linked to the bulk band crossings—are clearly resolved.

Beyond the topological properties, we further examine the WF characteristics of these electride systems. Owing to their weak confinement by atomic nuclei, exhibit enhanced surface mobility relative to conventional valence electrons, resulting in intrinsically low WFs. As shown in Figure 3e‐i, ii and Figures S11 and S12, both the bulk and corresponding monolayer structures exhibit ultralow WFs (with minimum values of 2.44 eV for bulk and 2.53 eV for monolayer), which are comparable to or even lower than those of known 2D inorganic electrides, such as Y_2_C (2.84 eV) [8], Sc_2_C (3.78 eV) [43], and Ca_2_N (2.6 eV) [9]. Moreover, after passivating the monolayer surfaces with hydrogen atoms to neutralize the IAEs, a significant increase in WF is consistently observed, further confirming that the low WFs in inorganic electrides are closely linked to the presence of IAEs (see Figure 3e‐i, iii; Figure S13). Leveraging this intrinsic property, we subsequently explored their potential applications in catalytic NH_3_ synthesis.

### 2.4 2D Inorganic Electride Loaded With Ru Promotes NH_3_ Synthesis

**FIGURE 4:**
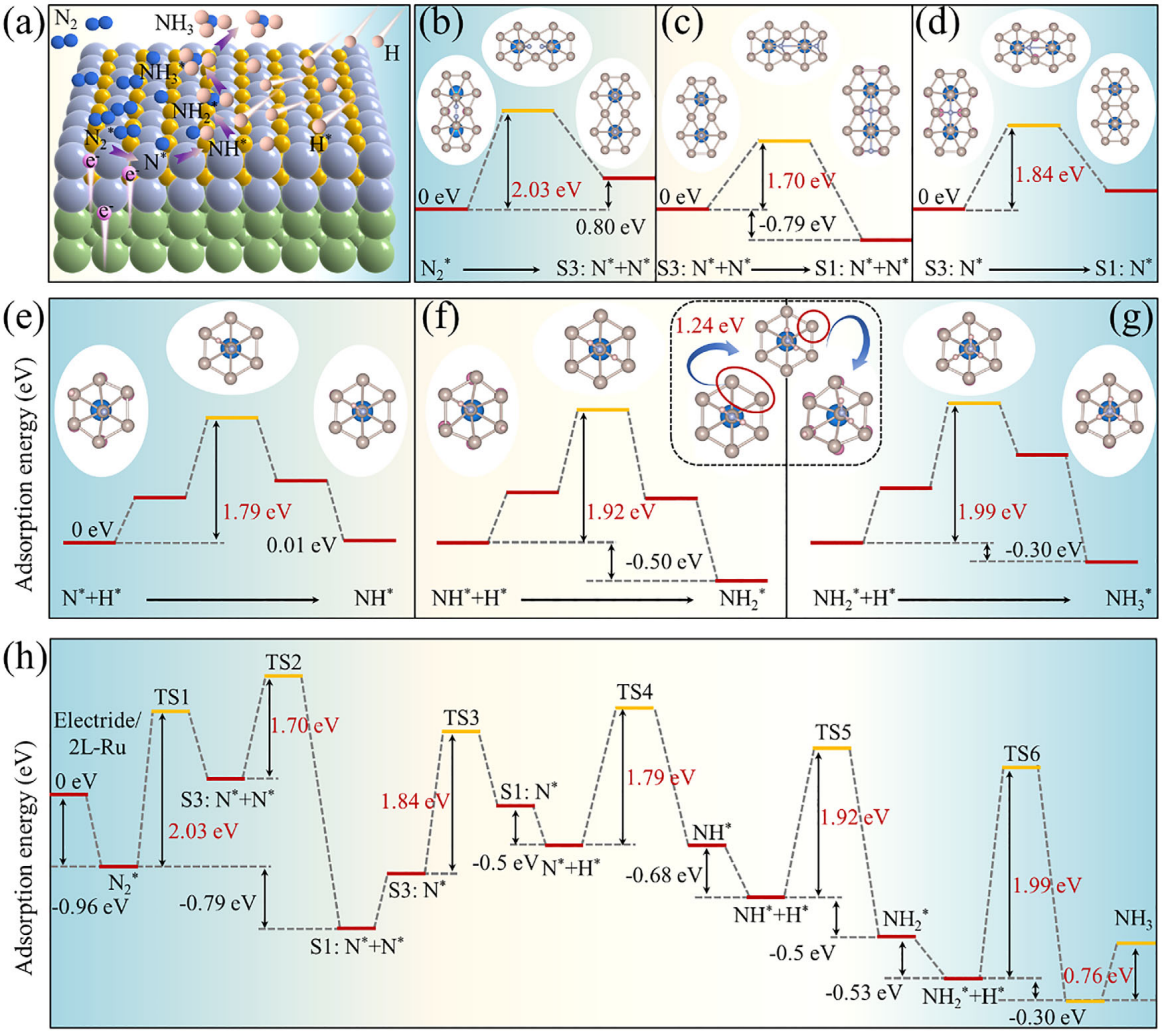
(a) Schematic diagram of Ru‐loaded monolayer inorganic electrides AB in the NH_3_ synthesis reaction. (b, c) show the N_2_ dissociation pathway and the associated energy barrier on the Ru/SrCu surface, respectively. (d) Present the energy barrier for the transition of an N atom from its stable adsorption configuration to a metastable configuration on the Ru/SrCu surface. (e–g) show energy profiles for three adsorbed intermediates (e) NH, (f) NH_2_, and (g) NH_3_ on the Ru/SrCu surface. (h) shows energy profiles of the reaction path for the NH_3_ synthesis on the Ru/SrCu surface.

Currently, transition metal‐based catalysts have attracted considerable attention in the field of NH_3_ synthesis, with hexagonally‐structured Ru metal emerging as a representative example. However, hydrogen poisoning has long hindered the further enhancement of the catalytic performance of isolated Ru metal. Notably, inorganic electrides—owing to their exceptional reversible hydrogen storage capacity and strong electron‐donating properties—provide a promising material platform for designing high‐performance NH_3_ synthesis catalysts, as shown in Figure 4a. More recently, various inorganic electride‐supported Ru catalysts [44, 45, 46, 47, 48, 49] (e.g., Ru/LaCoSi [45] and Ru/C12A7 [44, 47]) and isolated inorganic electrides [29, 50, 51, 52] (e.g., LaRuSi [50] and CeH_2_ [29]) have been demonstrated to exhibit unique catalytic advantages in NH_3_ synthesis. Nevertheless, existing research remains predominantly focused on bulk inorganic electride systems, while studies on their monolayer structures are still relatively scarce.

Furthermore, in various catalytic reactions, the WF is widely recognized as a critical descriptor for enhancing surface reactivity, particularly in NH_3_ synthesis, where it significantly promotes the dissociation of nitrogen molecule (N_2_). Remarkably, inorganic electrides—emerging as promising catalysts for NH_3_ synthesis—possess IAEs characterized by weak nuclear binding. Compared to conventional orbital electrons, these IAEs can be more readily excited to the material surface (exhibiting strong electron‐donating properties), thereby effectively facilitating N_2_ (N ≡ N) activation. As confirmed in Figure 3e, the ultralow WF of such systems originates from the IAEs, which resembles 2DEG behavior. To further explore the application potential of such systems in NH_3_ synthesis, we selected the noble‐metal‐free 2[SrCu]^+^:2e^−^ as the substrate and systematically investigated its performance after loading with Ru metal (Ru/2[SrCu]^+^:2e^−^), as shown in Figure 4 and discussed in Section S8.

Through an analysis of the adsorption energy and activated states of N_2_ at various Ru/2[SrCu]^+^:2e^−^ sites, one can find that horizontal adsorption at the Ru‐bridge site is the most favorable configuration, as shown in Figures S14 and S15. Furthermore, based on an examination of all possible adsorption configurations of the two N atoms on the Ru/2[SrCu]^+^:2e^−^ surface (see Figure S16), the dissociation of N_2_ proceeds via a two‐step mechanism (see Figure 4b,c): the first step involves the cleavage of the N≡N bond to form a metastable intermediate state, which requires overcoming an energy barrier of 2.03 eV; the second step consists of the migration of the resulting N atoms to two Ru‐bridge sites—the most stable configuration—associated with an energy barrier of 1.70 eV. Notably, the energy barriers for both steps are comparable to the N_2_ dissociation barrier on isolated Ru metal (2.01 eV) [45]. Compared to other Ru‐loaded inorganic electrides (such as Ru/C12A7: 49 kJ/mol [44], Ru/LaCoSi family: < 1 eV [45]), the Ru/2[SrCu]^+^:2e^−^ catalyst does not show the expected catalytic advantage. However, when N_2_ adsorbs horizontally at the non‐optimal Ru‐top site, it can directly dissociate into two N atoms via a one‐step reaction (see Figure S17). The dissociation energy barrier of this pathway is merely 1.65 eV, which is significantly lower than the rate‐determining barrier of the bridge‐site pathway and also superior to the N_2_ dissociation barrier on isolated Ru metal. Remarkably, comparing the two initial adsorption configurations, the strong adsorption of N_2_ at the Ru‐bridge site induces far more severe lattice distortion of the Ru‐layer than the weak adsorption at the Ru‐top site, leading to a higher reaction barrier for N_2_ dissociation via the bridge‐site pathway.

Furthermore, on the surface of pure Ru metal, hydrogen molecule (H_2_) dissociates through a nearly barrierless pathway, and the resulting two H atoms exhibit a relatively strong adsorption energy (E_ad_ = −1.48 eV) [46], indicating pronounced H poisoning. In contrast, the Ru/2[SrCu]^+^:2e^−^ catalyst raises the H_2_ dissociation barrier to 0.23 eV and weakens the dihydrogen adsorption energy to −1.05 eV (*vs* −1.48 eV) (see Figure S18 and Table SIV). This higher dissociation barrier, together with weaker dihydrogen adsorption, promotes H desorption from the surface, thereby effectively alleviating H poisoning.

When a single N atom is adsorbed on the Ru/2[SrCu]^+^:2e^−^ surface, its most stable adsorption site is also located at the Ru‐bridge site (see Figure S19). However, due to the limited cavity around the neighboring Ru atoms, the Ru‐bridge site is unfavorable for the subsequent NH_3_ synthesis reaction. In contrast, although the Ru‐top and Ru‐hollow sites are not the most stable adsorption sites for the N atom, they can provide a more suitable local environment for the stepwise hydrogenation process leading to NH_3_ formation. Furthermore, compared with the Ru‐hollow sites on the Ru/2[SrCu]^+^:2e^−^ surface, NH_3_ possesses a more stable adsorption energy on the Ru‐top site. (see Figure S20). Therefore, to transition from a stable adsorption site to an efficient reaction pathway, the N atom needs to migrate from the Ru‐bridge site to the Ru‐top site. This diffusion process entails overcoming an energy barrier of 1.84 eV, as shown in Figure 4d. In addition, the most stable adsorption site for H atoms on the Ru/2[SrCu]^+^:2e^−^ surface is located at the Ru‐hollow site (see Figure S21).

Based on the above analysis, we took the initial configuration of N atoms adsorbed at the Ru‐top site and the Ru‐hollow site as the hydrogenation selection to study the three reaction steps of NH_3_ synthesis: N + H → NH, NH + H → NH_2_, and NH_2_ + H → NH_3_ (NH, NH_2_, and NH_3_ represent three adsorbed intermediates), as shown in Figure 4e–g. Notably, on the Ru/2[SrCu]^+^:2e^−^ surface, the configuration of NH_2_ with two H atoms separated by one Ru atom is more favorable for NH_3_ formation. Therefore, the NH_2_ generated in the second step requires overcoming an energy barrier of 1.24 eV to transition into the NH_2_ configuration of the third step (see inset of Figure 4f). Additionally, the energy of the NH_2_ in the initial state of the third step is lower than that in the final state of the second step (see Figure S22). Finally, the calculated results indicate that these three reaction processes require overcoming energy barriers of 1.79, 1.92, and 1.99 eV, respectively, from the initial state to the final state, which is comparable to the performance of some existing bulk inorganic electrides (see Table SIV) (e.g., Er_5_Si_3_: 0.97, 1.26, 1.75 eV; Ru/LaMnSi: 1.22, 1.73, 1.39 eV; LaRuSi: 1.47, 1.61, 1.72 eV) [6, 45, 50]. Figure 4h and Figure S23 show the complete free energy curve and corresponding energy barriers for the NH_3_ synthesis on the Ru/2[SrCu]^+^:2e^−^ surface. These results indicate that this system holds promise as a potential candidate for 2D inorganic electrides‐supported Ru metal in promoting NH_3_ synthesis.

## 3 Conclusion

In conclusion, guided by established design principles for 2D inorganic electrides, eight AB‐type 2D inorganic electrides structurally analogous to Ca_2_N have been designed by leveraging the electronegativity difference between main‐group II‐A elements (A: Ca, Sr, Ba) and TMs B (B: Cu, Ag, Au). In these systems, the main‐group II‐A elements function as cations, whereas the TMs adopt anionic roles. Orbital electron transfer analysis reveals that each main group‐II A atoms contributes two valence *s*‐electrons: one is transferred to the *s*‐orbital of the TMs, completing an *s^2^* configuration; the other localizes in the interlayer region, forming a 2D IAE layer confined between atomic sheets. Notably, even in monolayer form, these structures retain electride features, with the IAEs floating on the surface, constituting a quasi‐2DEG. Moreover, these monolayer systems exhibit significant magnetic responses attributable to the surface‐floating IAEs. Analysis of the magnetic ground states reveals that five systems exhibit FM ordering, two display AFM behavior, and one remains NM. Upon passivation of the surface IAEs with hydrogen atoms, all systems transition to an NM state, further confirming that the observed magnetism originates exclusively from the surface IAEs. More importantly, these systems exhibit diverse topological phases and ultralow WFs (Φ_WF_ = 2.58–2.91 eV). Using SrCu as a representative system, supporting Ru metal leads to promising catalytic activity for NH_3_ synthesis. These findings not only expand the family of 2D inorganic electrides but also open new research avenues in spintronics and energy conversion.

## Author Contributions

**Xia Cheng**: investigation, formal analysis, data curation, visualization. **Zhenzhou Guo**: investigation. **Ying Yang**: formal analysis, investigation, conceptualization. **Weizhen Meng**: conceptualization, writing – review and editing, supervision, funding acquisition, investigation. **Weiyao Jia**: conceptualization, formal analysis, investigation. **Zhenxiang Cheng**: conceptualization, investigation, software. **Qianwen Zhang**: writing – original draft, formal analysis, investigation, visualization, data curation.

## Conflicts of Interest

The authors declare no conflicts of interest.

## Supporting information




**Supporting file**: advs76098‐sup‐0001‐SuppMat.docx

## Data Availability

The data that support the findings of this study are available on request from the corresponding author. The data are not publicly available due to privacy or ethical restrictions.
