# Supplementary material for: 2D Inorganic Electrides: Interstitial Electrons as Key Drivers of Multifunctional Properties and Applications
Source: Adv Sci (Weinh). 2026 Jun 12:e76098. Online ahead of print. doi: 10.1002/advs.76098 (PMC13336974; doi:10.1002/advs.76098)
Supplement: Supplementary file 1 — Supporting file: advs76098‐sup‐0001‐SuppMat.docx [file ADVS-9999-e76098-s001.docx]

***Supporting Information***

**2D Inorganic Electrides: Interstitial Electrons as Key Drivers of Multifunctional Properties and Applications**

Qianwen Zhang^1#^, Xia Cheng^1#^, Zhenzhou Guo^2^, Weiyao Jia^1*^, Ying Yang^3*^, Zhenxiang Cheng^2*^, Weizhen Meng^4*^

^1^ School of Physical Science and Technology, Southwest University, Chongqing 400715, China.

^2^ Institute for Superconducting and Electronic Materials, Faculty of Engineering and Information Sciences, University of Wollongong, Wollongong 2500, Australia.

^3^ College of Physics and Electronic Engineering, Chongqing Normal University, Chongqing 401331, China.

^4^ College of Physics, Hebei Key Laboratory of Photophysics Research and Application, Hebei Normal University, Shijiazhuang 050024, China.

^#^Qianwen Zhang and Xia Cheng contributed equally to this work.

^*^Correspondence: wyjia@swu.edu.cn;

[20131184@cqnu.edu.cn](mailto:20131184@cqnu.edu.cn);

[cheng@uow.edu.au](mailto:cheng@uow.edu.au);

[mengweizhen@hebtu.edu.cn](mailto:mengweizhen@hebtu.edu.cn)

**Contents**

[S1. Calculation methods 2](#_Toc231299048)

[S2. Structural parameters of inorganic electrides AB 4](#_Toc231299049)

[S4. Phonon spectra and AIMD of monolayer and bulk inorganic electrides AB 6](#_Toc231299050)

[S5. Magnetic characteristics of monolayer AB, ABH and ABH_2_. 7](#_Toc231299051)

[S6. Electronic structures of monolayer and bulk inorganic electrides AB 10](#_Toc231299052)

[S7. Work functions of bulk AB and monolayer AB and ABH 12](#_Toc231299053)

[S8. NH_3_ synthesis of Ru/SrCu 13](#_Toc231299054)

**S1. Calculation methods**

To investigate the interstitial anion electrons (IAEs), magnetism, topological phases, work function and catalytic reaction of 2D inorganic electride AB (A=Ca/Sr/Ba, B=Cu/Ag/Au) family, we performed first-principles calculations based on the density functional theory (DFT), employing the Vienna ab initio Simulation Package ^[S1-S3]^. The exchange–correlation potential was treated using the generalized gradient approximation of the Perdew–Burke–Ernzerh (PBE) of functional ^[S4]^. The cutoff energy was set as 500 eV. The Brillouin zone of bulk and monolayer structures was sampled using a Monkhorst–Pack *k*-mesh of 9 × 9 × 2 and 8 × 8 × 1, respectively. For monolayer structures, the vacuum layer exceeds 25 Å. The energy convergence criterion for electronic self-consistency was set as 10^-6^ eV. To describe the localized *d-*orbital, we employed the DFT+U approach for Transition metals (U=3 eV). The topological edge state of monolayer SrCu were calculated based on the Wannier functions ^[S5, S6]^, realized by using the WANNIERTOOLS package and WANNIER90 code ^[S7-S10]^. The phonon spectrum for monolayer materials 2D inorganic electride AB was calculated using the method proposed by Han et al ^[S11]^.

Constrained by the cavity size effect, the IAEs in electrides induce Stoner instability, which drives the system to a lower-energy magnetic ground state ^[S12]^. Therefore, the magnetism of some electrides originates from IAEs, including the AB-type 2D inorganic electride we designed. In some previous works, such as, T. J. Kim et al. selected organic electrides such as Rb^+^(cryptand[2.2.2])e^-^, where the magnetism originates from IAEs, and used an approximate approach that assigns the magnetic contribution to the cations nearest to the IAEs to calculate the magnetic ground state ^[S13]^. Their computational results showed excellent agreement with experimental measurements. Subsequently, Lu et al. introduced this approximation into the inorganic electride Yb_5_Sb_3_, which similarly confirmed that this method has high universality ^[S14]^. For AB-type 2D inorganic electride, their magnetism originates from the 2D IAEs, a characteristic that enables us to employ the aforementioned approximate method to determine their magnetic ground state. Therefore, we only need to assign the corresponding magnetic configurations to the cations in the upper and lower layers (i.e., Ca/Sr/Ba).

For the NH_3_ synthesis simulations, the Ru/2[SrCu]^+^:2e^-^ substrate was used in a 2×2×1 supercell structure. Remarkably, in all structural optimizations and transition state searches, only the surface-exposed Ru, Sr and adsorbed atoms/molecules (H, N, N_2_, NH, NH_2_, NH_3_) were allowed to relax, while bottom-layer Cu and Sr atoms remained fixed. Besides, the calculation methods of adsorption energy: E_ad_ = E_slab+N2_ - E_N2_ - E_slab_, E_ad_ = E_slab+NH_ – E_NH_ - E_slab_, E_ad_ = E_slab+NH2_ - E_NH2_ - E_slab_, E_ad_ = E_slab+NH3_ - E_NH3_ - E_slab_, E_ad_ = E_slab+N_ - E_N_ - E_slab_, E_ad_ = E_slab+H_ - E_H_ - E_slab_, E_ad_ = E_slab+NH_ - E_slab+N-H_, E_ad_ = E_slab+NH2_ - E_slab+NH-H_, E_ad_ = E_slab+NH3_ - E_slab+NH2-H_. Among them, the energies of N_2_, H_2_, NH, NH_2_ and NH_3_ are -16.48 eV, -6.76 eV, -6.36 eV, -12.78 eV and -19.24 eV respectively.

Analysis of the potential experimental synthesis of AB-type 2D inorganic electrides (A = Ca/Sr/Ba, B = Cu/Ag/Au). Specifically, The AB-type 2D inorganic electrides are derived from the experimentally realized 2D inorganic electride BaCu. The expanded systems are composed of relatively safe and common elements and are expected to be synthesizable using technical routes similar to those employed for the 2D inorganic electride BaCu. As described in the work of Wan et al. ^[S15]^, Ba ingot and Cu powder were mixed in a 1:1 molar ratio, placed into an evacuated quartz tube, sintered at 600 °C, then cooled to 400 °C and held at this temperature for 10 hours. Subsequently, structural characterization of the sample was performed at room temperature using X-ray diffraction (XRD), scanning electron microscopy (SEM), and other techniques.

**S2. Structural parameters of inorganic electrides AB**

One can find that identified 2D inorganic electrides, the majority adopt AB-type or A_2_B-type structures and belong to the *R*$\tilde{3}$*m* space group (SG), while only a few exist as ternary compounds. Even when transition metals are involved, these materials typically exist in positive oxidation states (see Table SI). Fig. S1 presents the eight AB-types inorganic electrides with *P6_3_/mmc* SG, along with their corresponding structural parameters.

**Table SI.** Identified 2D inorganic electrides and their corresponding SGs.

| 2D inorganic electrides | SGs | 2D inorganic electrides | SGs | 2D inorganic electrides | SGs |
| --- | --- | --- | --- | --- | --- |
| Ca_2_N family ^[S16]^ | *R*$\tilde{3}$*m* | Na_2_Cl family ^[S21]^ | *R*$\tilde{3}$*m* | LaCl family ^[S20]^ | *R*$\tilde{3}$*m* |
| Y_2_C family ^[S17]^ | *R*$\tilde{3}$*m* | Cs_2_O_1-x_F_x_ ^[S22]^ | *R*$\tilde{3}$*m* | CrB/PrGa family ^[S21]^ | *Cmcm* |
| Pr_5_(CoB_3_)_2_ family ^[S18]^ | *R*$\tilde{3}$*m* | CaCl family ^[S21]^ | *R*$\tilde{3}$*m* | Hf_2_S family ^[S23]^ | *P6_3_/mmc* |
| Gd_2_O ^[S19]^ | *R*$\tilde{3}$*m* | Zr_2_N family ^[S20]^ | *P6_3_/mmc* | Ti_2_C family ^[S21]^ | *R*$\tilde{3}$*m* |
| Al_2_C family ^[S20]^ | *P-3m1* | Sc_2_N family ^[S20]^ | *R*$\tilde{3}$*m* | Ca_2_O family ^[S21]^ | *R*$\tilde{3}$*m* |


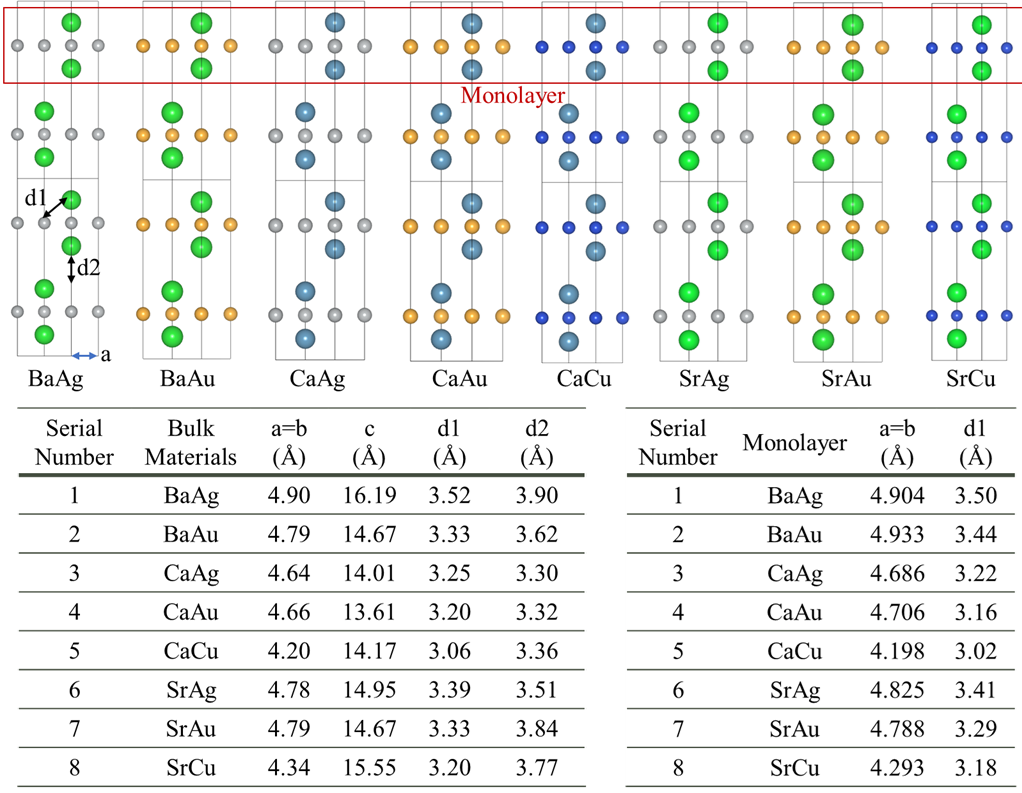


**Fig. S1.** Structural parameters of bulk and monolayer inorganic electrides AB.

**S3. The ELF of monolayer and bulk inorganic electrides AB**

**
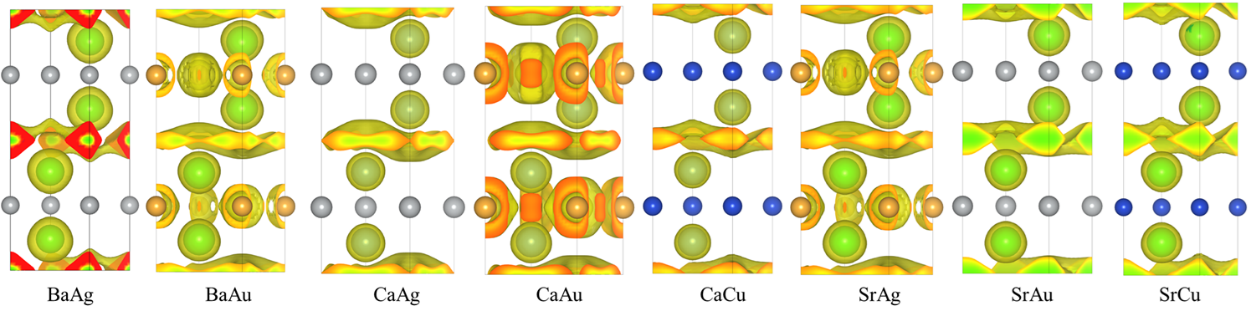
**

**Fig. S2.** The ELF of bulk inorganic electrides AB.

**
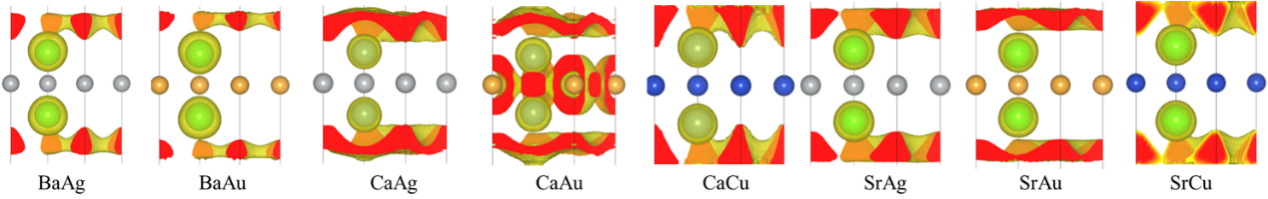
**

**Fig. S3.** The ELF of monolayer inorganic electrides AB.

**S4. Phonon spectra and AIMD of monolayer and bulk inorganic electrides AB**


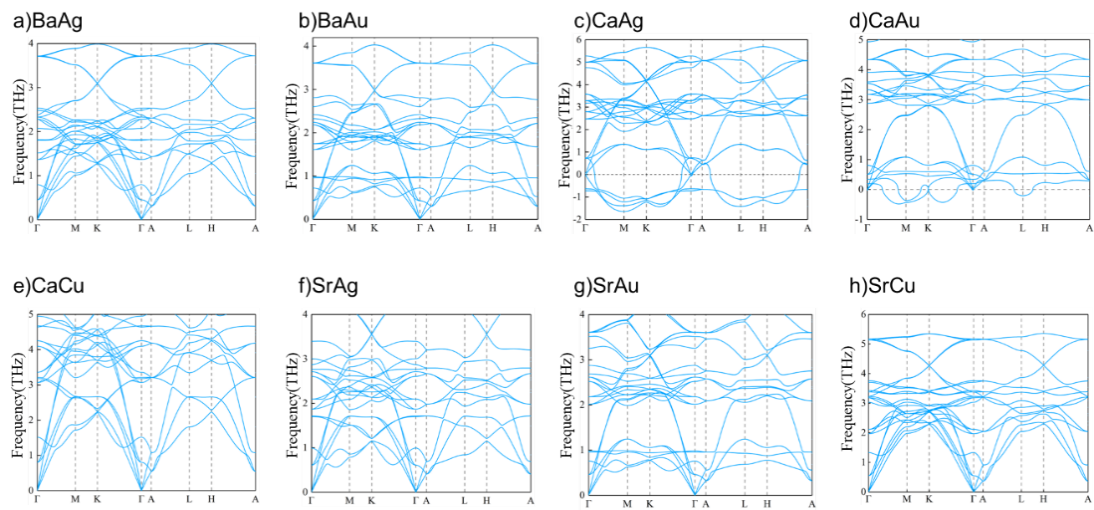


**Fig. S4. (a-h)** Phonon spectra of bulk inorganic electrides AB.


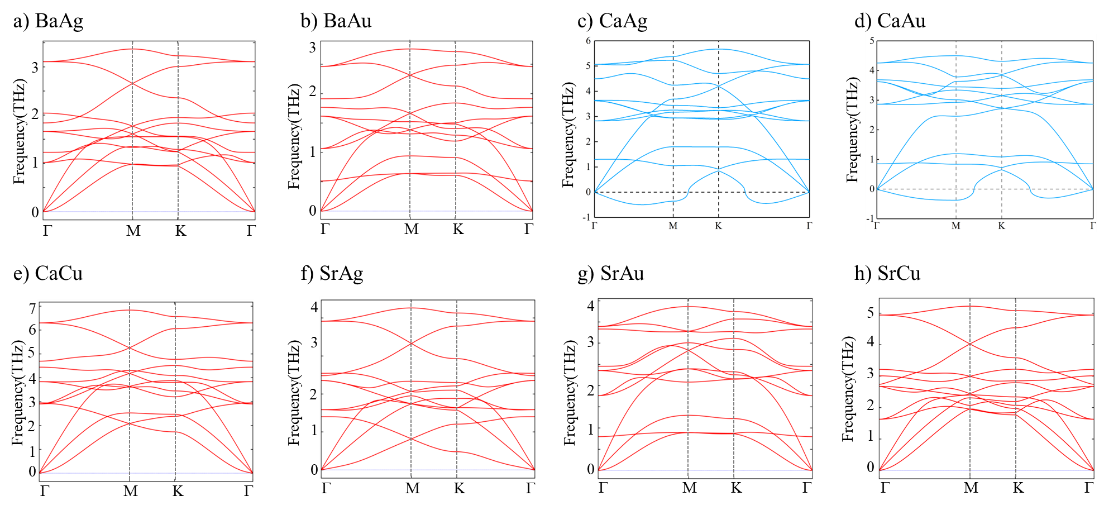


**Fig. S5. (a-h)** Phonon spectra of monolayer inorganic electrides AB.


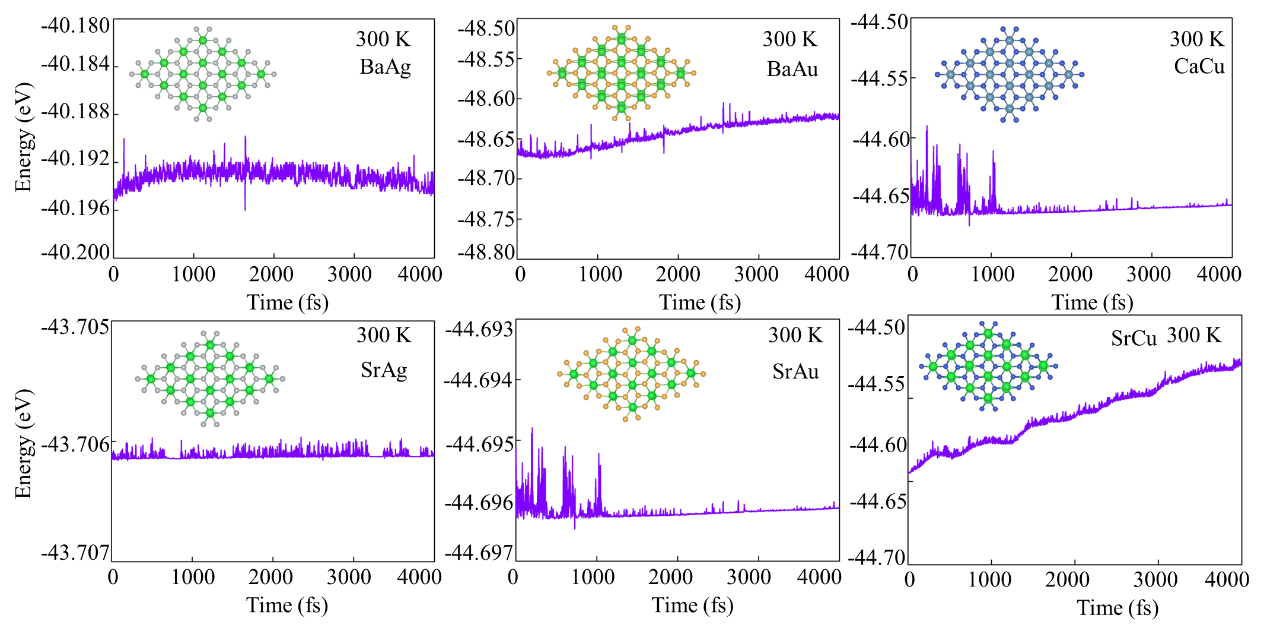


**Fig. S6.** Total energy variation of six 2D inorganic electrides in 300 K AIMD simulations with 3×3×1 supercells.

**S5. Magnetic characteristics of monolayer AB, ABH and ABH_2_.**

All eight monolayer candidate materials exhibit non-negligible magnetic moments under ferromagnetic (FM) ordering, with the magnetic moment contributed by interstitial anionic electrons (IAEs) playing a dominant role (see Table SII). Fig. S7 shows the magnetic ground states of AB and ABH structures under four magnetic configurations (FM, AFM1-3). Further analysis indicates that when the top-layer IAEs are hydrogenated, the system remains magnetism, but its magnetic moment is significantly reduced. When the bottom-layer IAEs are further hydrogenated, all systems transition to a non-magnetic (NM) state (see Table SIII).

**Table SII.** The total magnetic moment, atomic magnetic moment and interstitial anion electron magnetic moment of the monolayer AB under FM ordering.

**
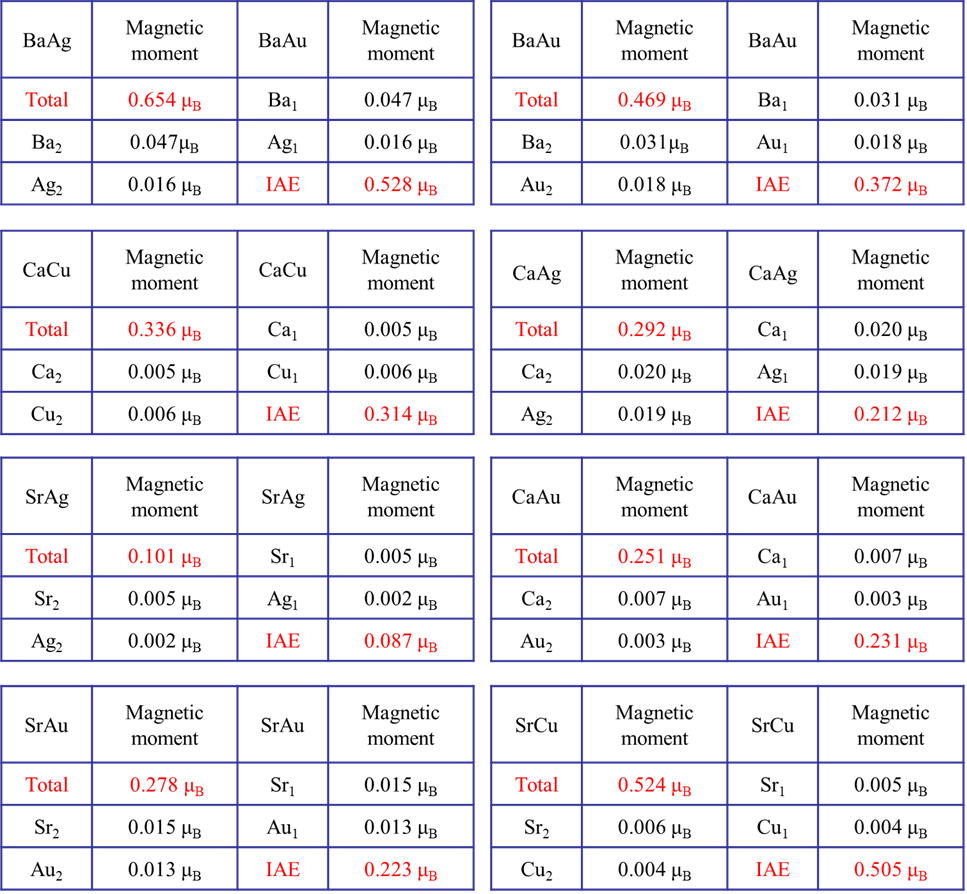
**

**Table SIII.** The total magnetic moment, atomic magnetic moment and ELF of the monolayer AB, ABH, and ABH_2_ under the magnetic ground state (MGS).

**
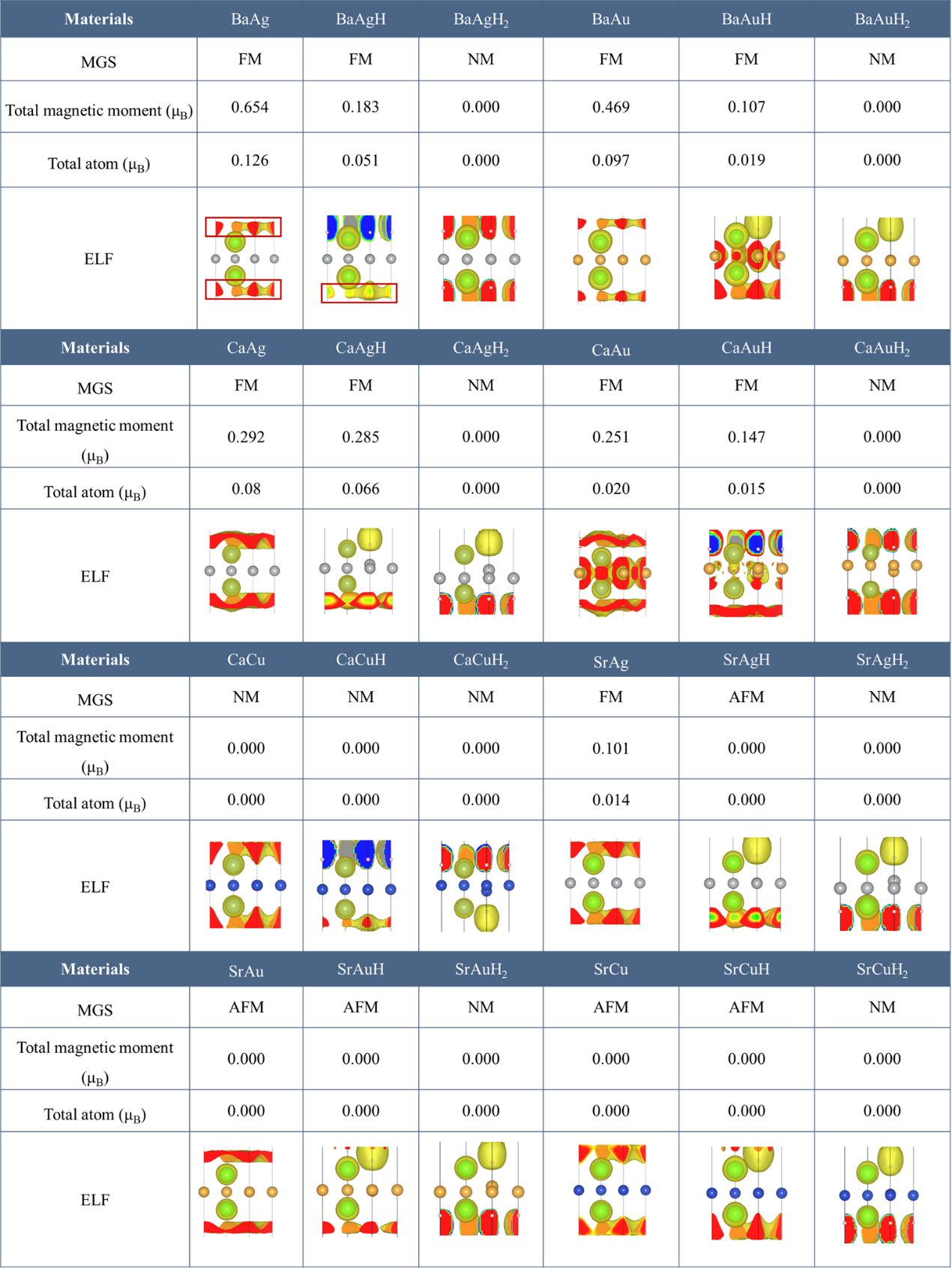
**

**
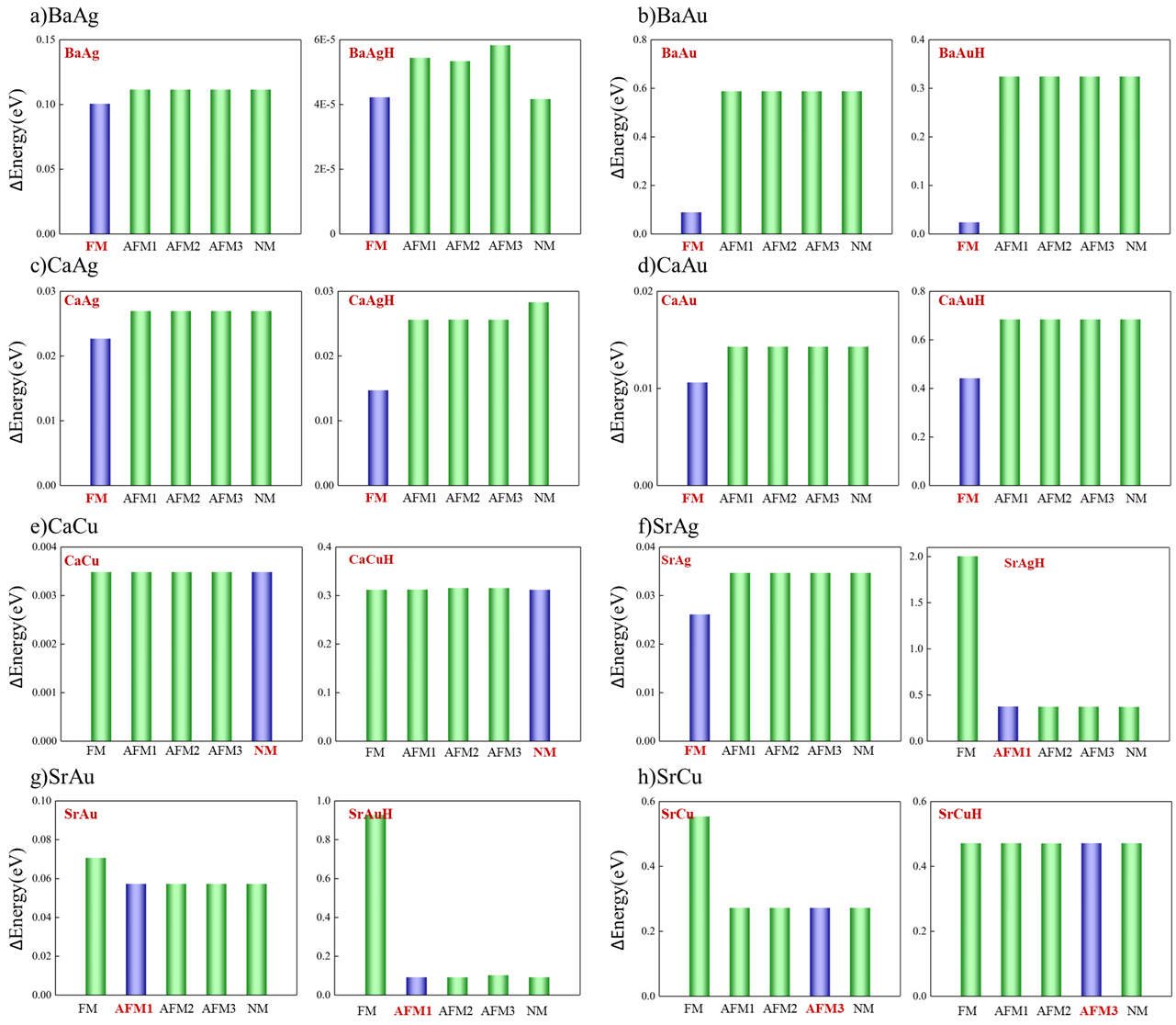
**

**Fig. S7. (a-h)** The magnetic ground states of AB and ABH structures under four magnetic configurations.

**S6. Electronic structures of monolayer and bulk inorganic electrides AB**

**
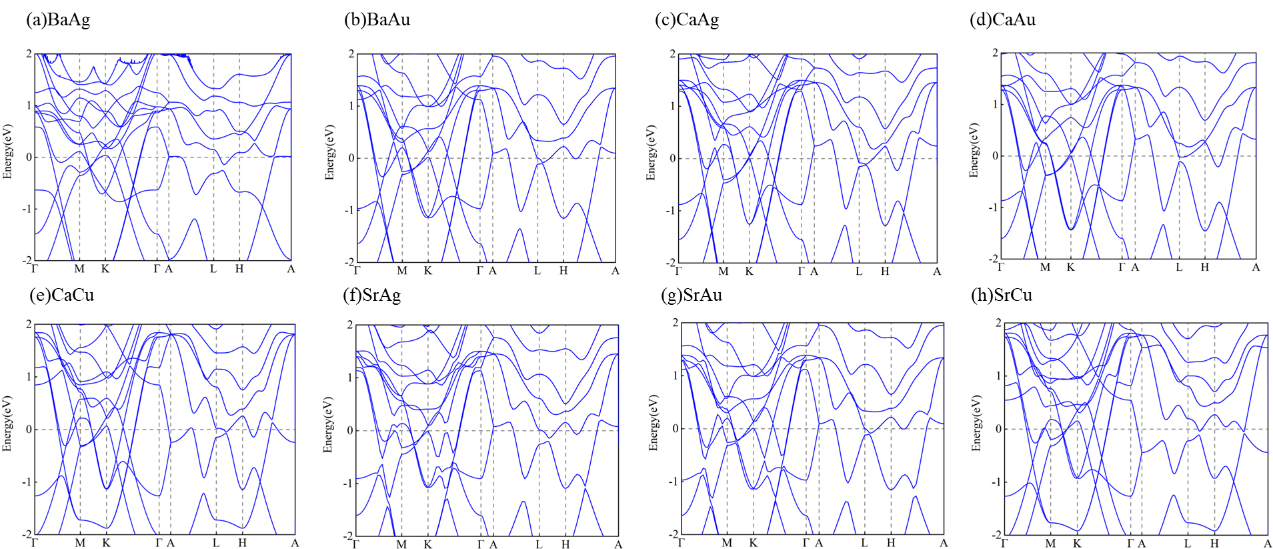
**

**Fig. S8. (a-h)** Electronic structures of bulk inorganic electrides AB.

**
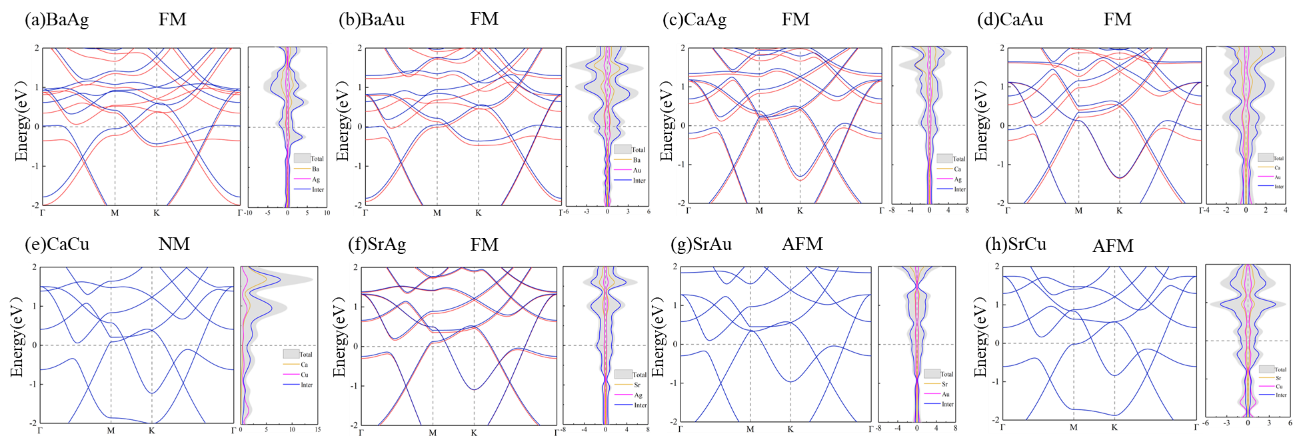
**

**Fig. S9.** **(a-h)** Electronic structures of monolayer inorganic electrides AB.

**
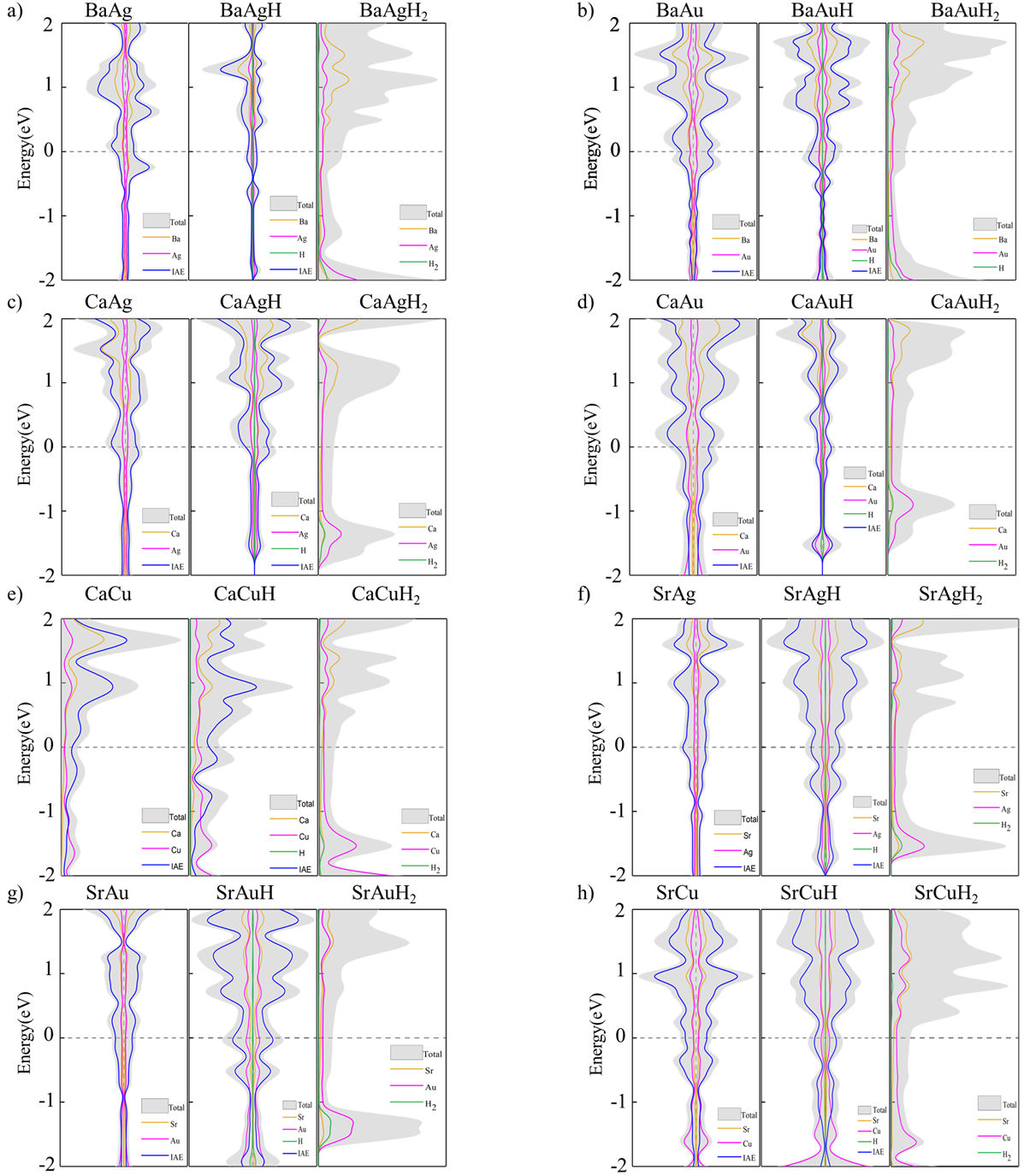
**

**Fig. S10.** **(a-h)** PDOS of monolayer AB, ABH, and ABH_2_ under stable magnetic ground states.

**S7. Work functions of bulk AB and monolayer AB and ABH**

**
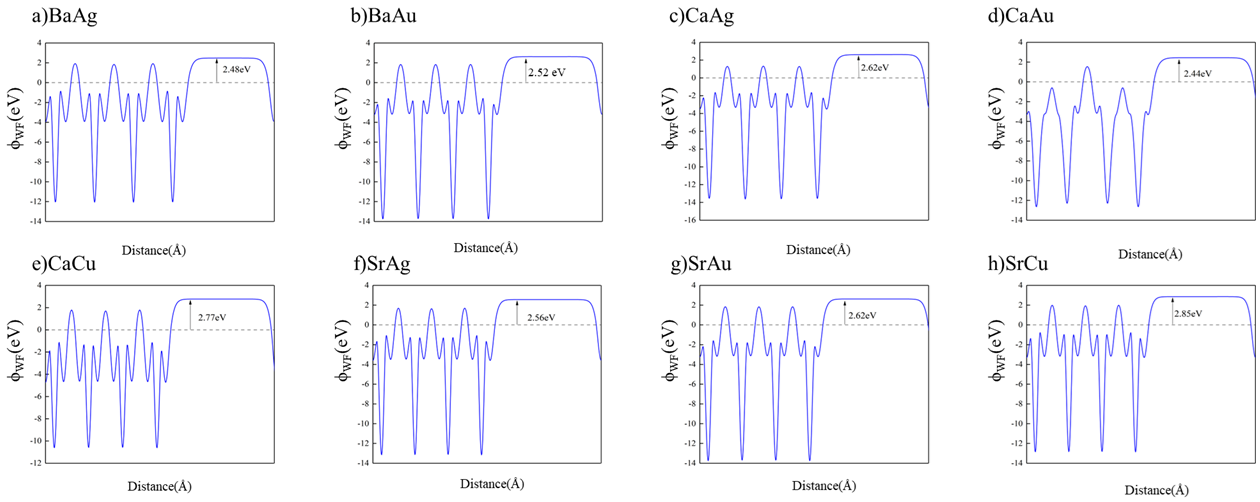
**

**Fig. S11. (a-h)** Work functions of bulk inorganic electrides AB.


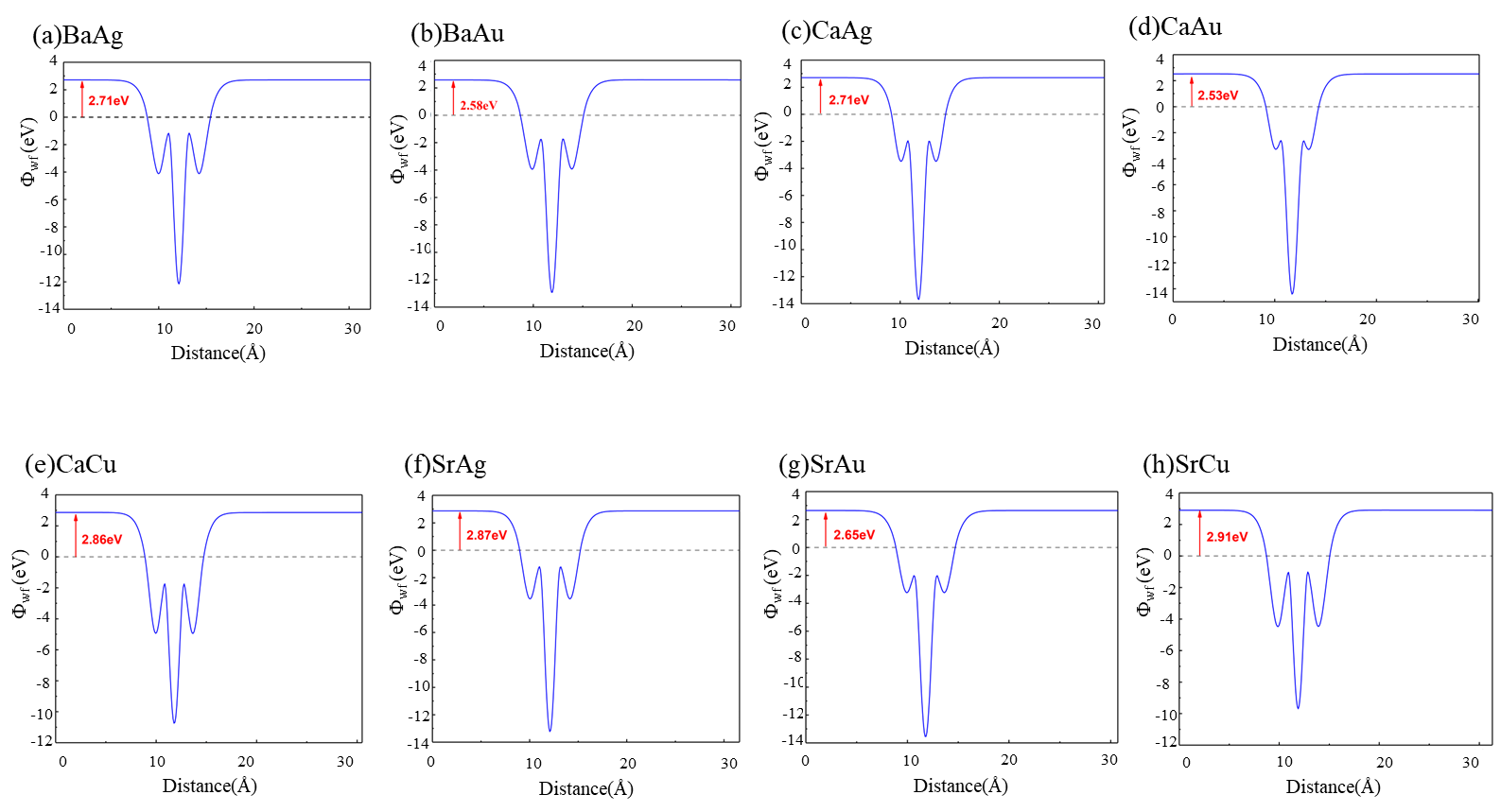


**Fig. S12. (a-h)** Work functions of monolayer inorganic electrides AB.


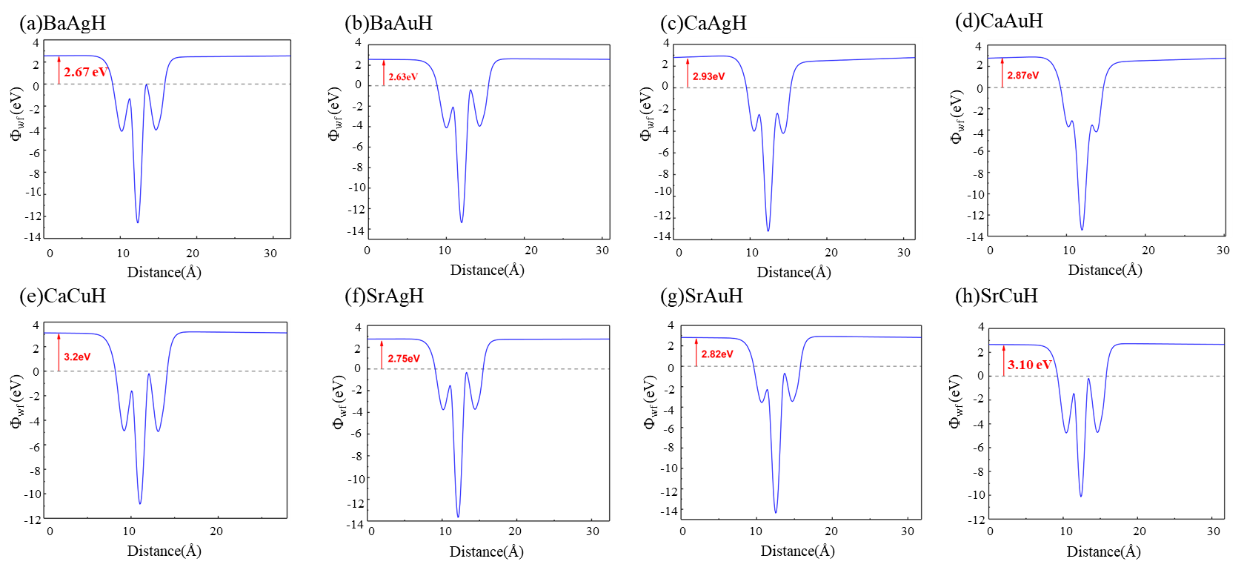


**Fig. S13. (a-h)** Work functions of monolayer inorganic electrides ABH.

**S8. NH_3_ synthesis of Ru/SrCu**

We loaded two layers of Ru metal on the surface of the monolayer inorganic electride SrCu. The first layer of Ru preferentially adsorbs at the IAEs site, located directly above the metal Cu atoms. The second layer of Ru adsorbs at the metal Sr site (see Figs. S14 (a, b)). Charge difference density (CDD) analysis reveals that electrons from the IAEs and orbital electrons from the metal Sr are transferred to the surface Ru layer, as shown in Fig. S14 (c).


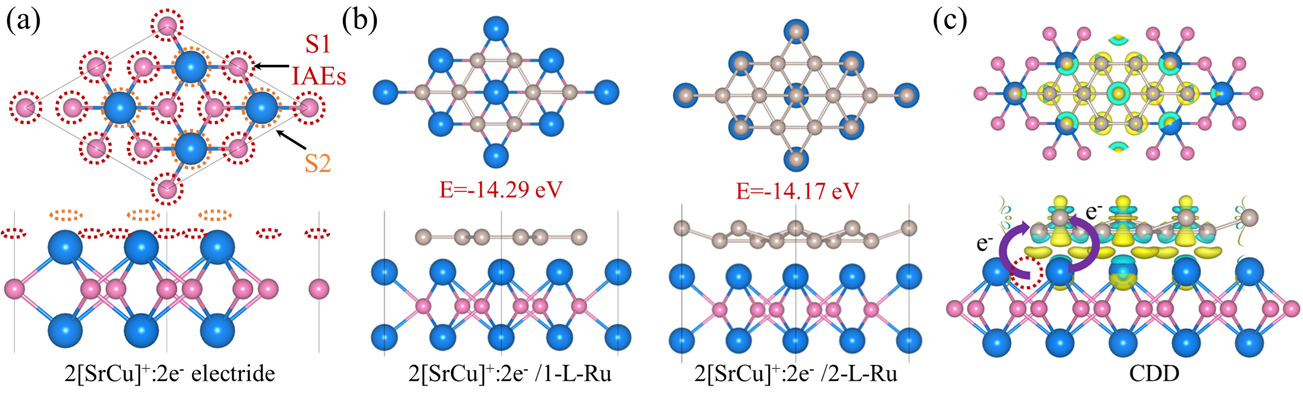


**Fig. S14. (a)** The adsorption sites of Ru atom on the monolayer SrCu surface. **(b)** The final optimized structures of Ru/SrCu. **(c)** Charge difference density (CDD) of Ru/SrCu.

When adsorbing N_2_ on the Ru/SrCu surface, we considered three adsorption sites, namely the S1-Ru-top site, the S2-Ru-hollow site, and the S3-Ru-bridge site. Considering the activation and adsorption energy of N_2_, the horizontally positioned N_2_ at the S3-Ru-bridge site is ultimately determined to be the optimal configuration (see Figs. S15 (a, b)).


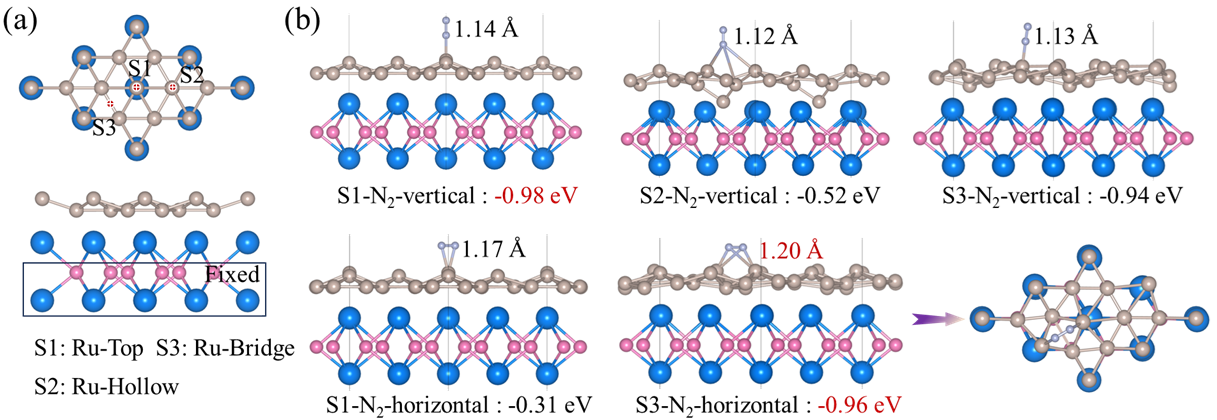


**Fig. S15. (a)** The adsorption sites of the monolayer Ru/SrCu surface. **(b)** The final optimized structures of N_2_ on the Ru/SrCu surface.

For the two N atoms, we considered six possible adsorption sites on the Ru/SrCu surface. The final optimization results indicate that the S4 site coincides with the S2 site, with the S2 site being the most stable adsorption configuration (see Figs. S16 (a, b)). However, as shown in Fig. S15, the dissociation of the N_2_ molecule (S3-Ru-bridge) into two N atoms at the S2 site requires passing through the S3 site as an intermediate metastable state (see Fig. S16 (c)).


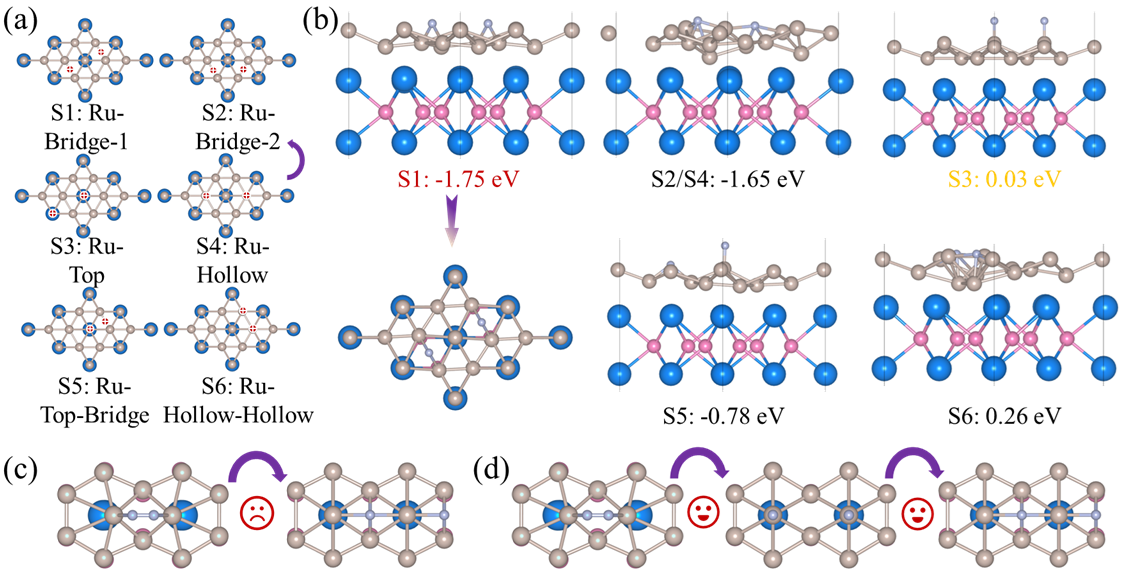


**Fig. S16. (a)** The adsorption sites of the monolayer Ru/SrCu surface. **(b)** The final optimized structures of two N atoms on the Ru/SrCu surface. **(c)** The process of N_2_ dissociation into two N atoms on the Ru/SrCu surface.


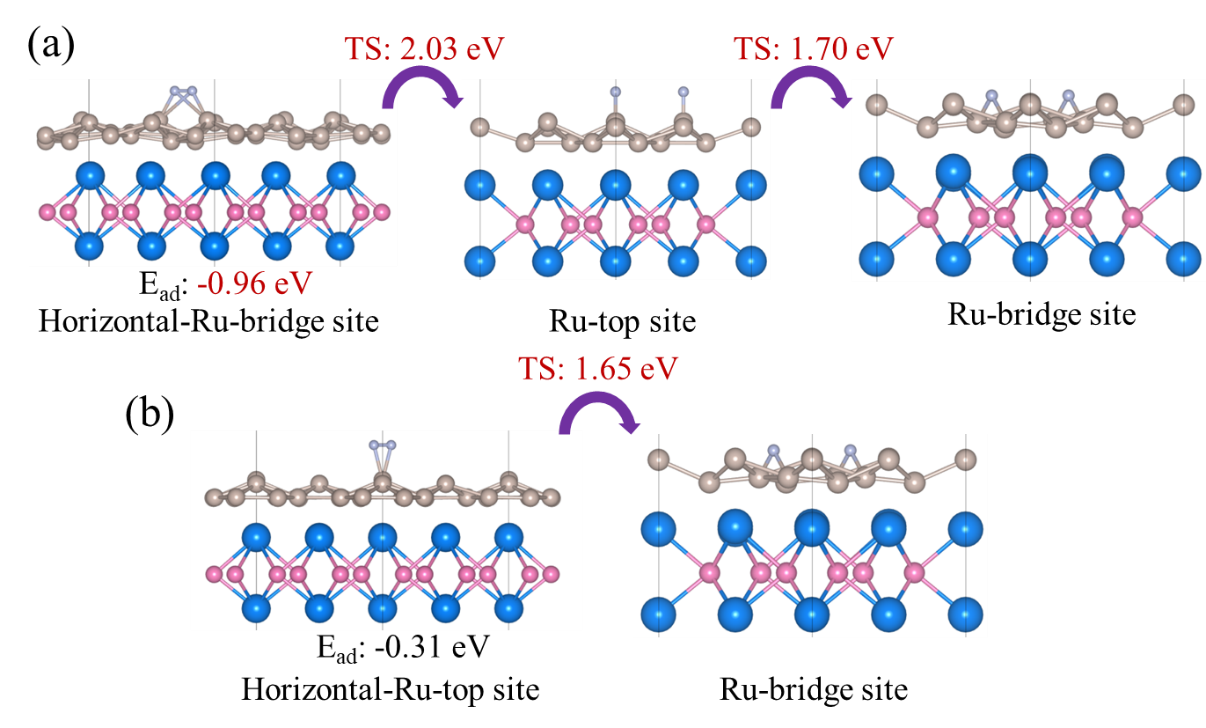


**Fig. S17.** (a) The initial and final states for N_2_ dissociation on the Ru-bridge site of the Ru/SrCu surface. (b) The initial and final states for N_2_ dissociation on the Ru-top site of the Ru/SrCu surface.


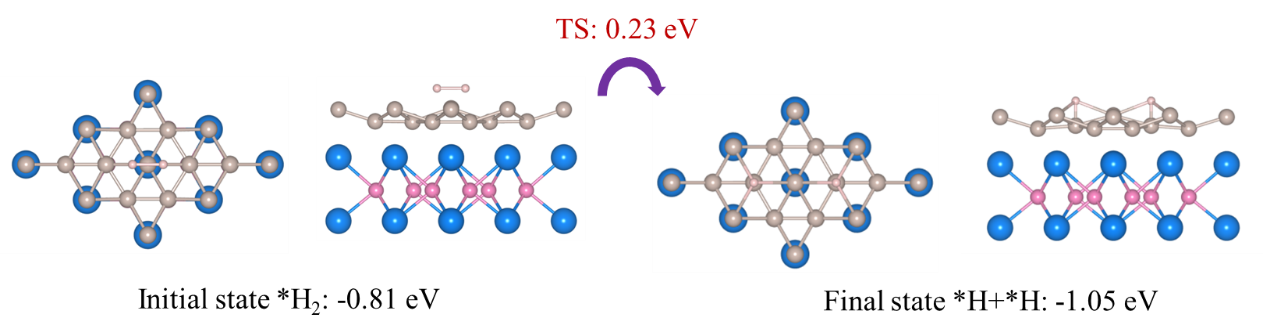


**Fig. S18.** Top and side structures of the initial and final states for H_2_ dissociation on the Ru/SrCu.


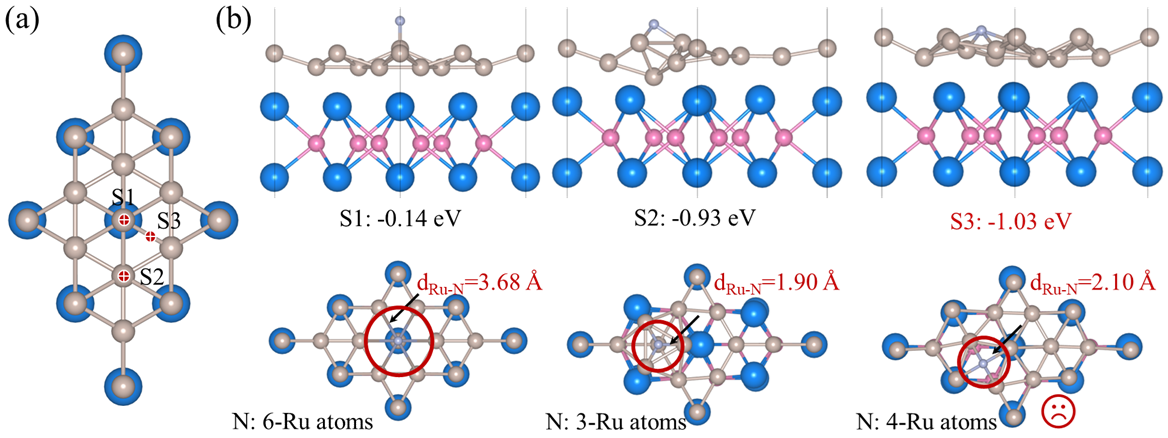


**Fig. S19. (a)** The adsorption sites of the monolayer Ru/SrCu surface. **(b)** The final optimized structures of one N atom on the Ru/SrCu surface.


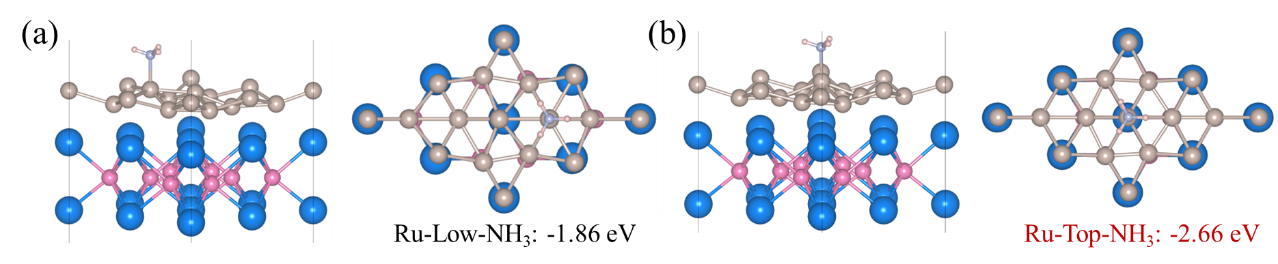


**Fig. S20. (a)** and **(b)** The final optimized structures of NH_3_ on the Ru-low and Ru-top sites of Ru/SrCu surface.


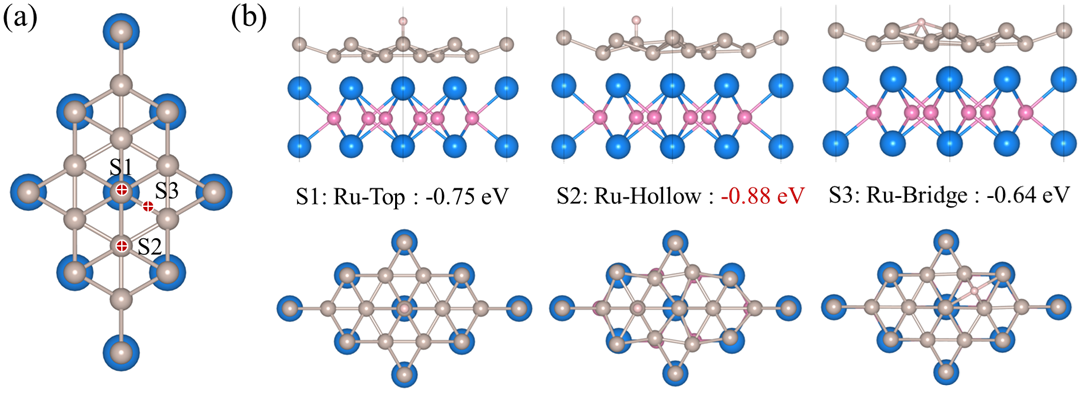


**Fig. S21. (a)** The adsorption sites of the monolayer Ru/SrCu surface. **(b)** The final optimized structures of one H atom on the Ru/SrCu surface.


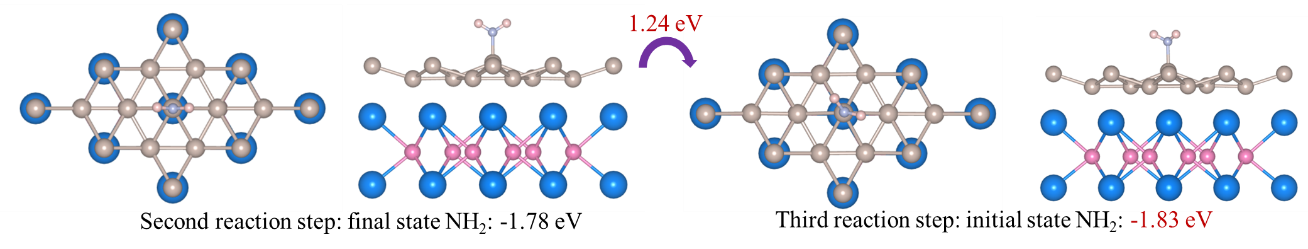


**Fig. S22. (a)** and **(b)** The adsorption energies corresponding to the two adsorption configurations of NH_2_ molecules on the Ru-top of the Ru/SrCu surface.


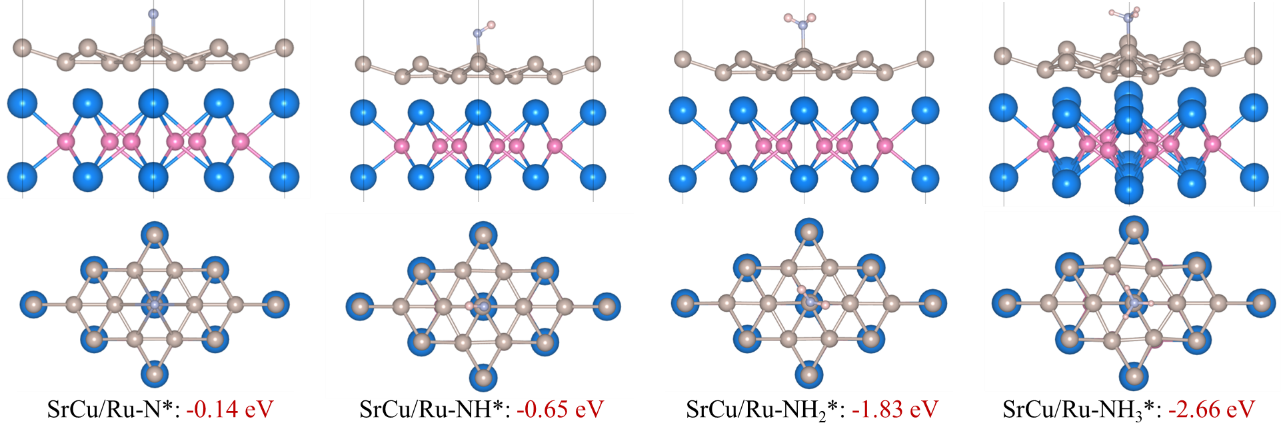


**Fig. S23.** The final optimized structures of N (S-1), NH, NH_2_, and NH_3_ on the Ru/SrCu surface.

Table SV. Adsorption energies of all intermediates and co-adsorbed species involved in ammonia synthesis over SrCu/Ru surface, including two distinct adsorption sites (S-1 and S-3) for atomic N.

| SrCu (*) + atoms/molecules | E_ads_ (eV) | SrCu (*) + atoms/molecules | E_ads_ (eV) |
| --- | --- | --- | --- |
| N_2_* | -0.96 | NH_2_*+H* | -2.36 |
| S-3 N* | -1.03 | NH_3_* | -2.66 |
| S-1 N* | -0.14 | H_2_* | -0.81 |
| H* | -0.88 | H*+H* | -1.05 |
| N* + H* | -0.64 | N*+N* | -1.75 |
| NH* | -0.65 |  |  |
| NH*+H* | -1.33 |  |  |
| NH_2_* | -1.83 |  |  |

Table SIV. Comparison of NH_3_ synthesis reaction energy barriers between the Ru/SrCu in this work and reported inorganic electrides, electride-supported Ru and pure Ru catalysts. References [6], [29], [45], [46], [50] are cited in the main text.

| Materials | TS: N_2_→N+N | TS1: N+H→NH | TS2: NH+H→NH_2_ | TS3: NH_2_+H→NH_3_ |
| --- | --- | --- | --- | --- |
| This work | 2.03/1.70 eV | 1.79 eV | 1.92 eV | 1.99 eV |
| Ru ^[46]^ | 2.01 eV | 1.15 eV | 1.26 eV | 1.24 eV |
| LaRuSi ^[50]^ | 0.93 eV | 1.47 eV | 1.61 eV | 1.72 eV |
| CaRuSi ^[50]^ | 1.21 eV | 1.76 eV | 1.55 eV | 1.75 eV |
| Y_5_Si_3_ ^[S24]^ | 0.78 eV | 0.89 eV | 1.05 eV | 1.65 eV |
| Y_5_Ge_3_ ^[S24]^ | 1.17 eV | 0.79 eV | 0.88 eV | 1.29 eV |
| Er_5_Si_3_ ^[6]^ | 0.88 eV | 0.97 eV | 1.26 eV | 1.75 eV |
| CeH_2_ ^[29]^ | 2.10 eV | 1.20 eV | 1.40 eV | 2.20 eV |
| Ru/LaCoSi ^[45]^ | 0.74 eV | 1.07 eV | 1.45 eV | 1.26 eV |
| Ru/LaFeSi ^[45]^ | 0.62 eV | 1.16 eV | 1.62 eV | 1.28 eV |
| Ru/LaMnSi^[45]^ | 0.61 eV | 1.22 eV | 1.73 eV | 1.39 eV |

**References**

[S1] Kresse G.; Hafner J. Ab initio molecular-dynamics simulation of the liquid-metal–amorphous-semiconductor transition in germanium. *Phys. Rev. B.* **1994**, *49*, 14251.

[S2] Kresse G.; Furthmüller J. Efficient iterative schemes for ab initio total-energy calculations using a plane-wave basis set. *Phys. Rev. B.* **1996**, *54*, 11169.

[S3] Blöchl P E. Projector augmented-wave method. *Phys. Rev. B.* **1994**, *50*, 17953.

[S4] Perdew J P.; Burke K.; Ernzerhof M. Ernzerhof. Generalized gradient approximation made simple. *Phys. Rev. Lett.* **1996**, *77*, 3865.

[S5] Marzari N.; Vanderbilt D. Maximally localized generalized Wannier functions for composite energy bands. *Phys. Rev. B.* **1997**, *56*, 12847.

[S6] Souza I, Marzari N. Vanderbilt D. Maximally localized Wannier functions for entangled energy bands. *Phys. Rev. B.* **2001**, *65*, 035109.

[S7] Wu Q S.; Zhang S N, Song H F. et al. WannierTools: An open-source software package for novel topological materials. *Comput. Phys. Commun*. **2018**, *224*, 405.

[S8] Sancho M P L.; Sancho J M L.; Rubio J. Quick iterative scheme for the calculation of transfer matrices: application to Mo (100). *J. Phys. F: Met. Phys*. **1984**, *14*, 1205.

[S9] Sancho M P L.; Sancho J M L.; Sancho J M L. et al. Highly convergent schemes for the calculation of bulk and surface Green functions. *J. Phys. F: Met. Phys*. **1985**, *15*, 851.

[S10] Mostofi A. A.; Yates J R.; Lee Y S. et al. Marzari. wannier90: A tool for obtaining maximally-localised Wannier functions. *Comput. Phys. Commun*. **2008**, *178*, 685.

[S11] Han X.-Q.; Guo P.-J.; Gao Z.-F.; Lu Z.-Y. PhononBench:A Large-Scale Phonon-Based Benchmark for Dynamical Stability in Crystal Generation. arXiv:2512.21227.

[S12] Sui X.; Wang J. Duan; W. Prediction of Stoner-Type Magnetism in Low-Dimensional Electrides. *J. Phys. Chem. C* **2019**, 123(8), 5003-5009.

[S13] Kim T. J.; Yoon H.; Han M J. Calculating magnetic interactions in organic electrides.  *Phys. Rev. B.* **2018**, *97*, 214431.

[S14] Lu Y.; Wang J.; Li J. et al. Realization of Mott-insulating electrides in dimorphic Yb_5_Sb_3_. *Phys. Rev. B.* **2018**, *98*, 125128.

[S15] Wan B.; Yuan Y.; Zheng L. et al. BaCu, a two-dimensional electride with Cu anions. *J. Am. Chem. Soc.*, **2024**, 146(25), 17508-17516.

[S16] Novoselov D Y.; Korotin D M.; Shorikov A O. et al. Interacting electrons in two-dimensional electride Ca_2_N. *J. Phys. Chem. C,* **2021**, *125*, 15724-15729.

[S17] Zhang X.; Xiao Z.; Lei H. et al. Two-dimensional transition-metal electride Y_2_C. *Chem. Mater*., **2014**, *26*, 6638-6643.

[S18] Zhu Q.; Frolov T.; Choudhary K. Computational discovery of inorganic electrides from an automated screening. *Matter,* **2019**, *1*, 1293-1303.

[S19] Liu S.; Wang C.; Jeon H. et al. Interlayer exchange interaction driven topological phase transition in antiferromagnetic electride Gd_2_O. *Phys. Rev. B,* **2022**, *105*, L041406.

[S20] Zhang Y.; Wang H.; Wang Y. et al. Computer-assisted inverse design of inorganic electrides. *Phys. Rev. X,* **2017**, *7*, 011017.

[S21] Ming W.; Yoon M.; Du M H. et al. First-principles prediction of thermodynamically stable two-dimensional electrides. *J. Am. Chem. Soc.,* **2016**, *138*, 15336-15344.

[S22] Tada T.; Takemoto S.; Matsuishi S. et al. High-throughput ab initio screening for two-dimensional electride materials. *Inorg. Chem.,* **2014**, *53*, 10347-10358.

[S23] Kang S H.; Thapa D.; Regmi B. et al. Chemically stable low-dimensional electrides in transition metal-rich monochalcogenides: theoretical and experimental explorations. *J. Am. Chem. Soc.,* **2022**, *144*, 4496-4506.

[S24] Cao Y.; Liu P.; Li J.; Wang J.; Sun Y.; Cheng X. Microscopic Mechanism of Enhanced Catalytic Activity for Ammonia Synthesis in Y_5_M_3_ (M = Si/Ge) Electrides. *J. Phys. Chem. C* **2023**, 127, 6, 2953–2962.
